# A randomised phase II trial of temozolomide with or without cannabinoids in patients with recurrent glioblastoma (ARISTOCRAT): protocol for a multi-centre, double-blind, placebo-controlled trial

**DOI:** 10.1186/s12885-023-11792-4

**Published:** 2024-01-15

**Authors:** Divyalakshmi Bhaskaran, Joshua Savage, Amit Patel, Fiona Collinson, Rhys Mant, Florien Boele, Lucy Brazil, Sara Meade, Peter Buckle, Siân Lax, Lucinda Billingham, Susan C. Short

**Affiliations:** 1 School of Medicine, University of Leeds, LS2 9JT Leeds, UK; 2 Leeds Teaching Hospitals NHS Trust, Leeds, UK; 3 Cancer Research UK Clinical Trials Unit (CRCTU), Institute of Cancer & Genomic Sciences, University of Birmingham, Birmingham, UK; 4 Guy’s and St Thomas’ NHS Foundation Trust, London, UK; 5 University Hospitals Birmingham Foundation Trust, Birmingham, UK; 6 The Brain Tumour Charity, Hampshire, UK

**Keywords:** Randomised phase II trial, Glioblastoma, Cannabinoids, Sativex, Nabiximols, Brain cancer, Tumour, Temozolomide, Progression free survival, Overall survival, Adverse events

## Abstract

**Background:**

Glioblastoma (GBM) is the most common adult malignant brain tumour, with an incidence of 5 per 100,000 per year in England. Patients with tumours showing O^6^-methylguanine-DNA methyltransferase (MGMT) promoter methylation represent around 40% of newly diagnosed GBM. Relapse/tumour recurrence is inevitable. There is no agreed standard treatment for patients with GBM, therefore, it is aimed at delaying further tumour progression and maintaining health-related quality of life (HRQoL). Limited clinical trial data exist using cannabinoids in combination with temozolomide (TMZ) in this setting, but early phase data demonstrate prolonged overall survival compared to TMZ alone, with few additional side effects. Jazz Pharmaceuticals (previously GW Pharma Ltd.) have developed nabiximols (trade name Sativex®), an oromucosal spray containing a blend of cannabis plant extracts, that we aim to assess for preliminary efficacy in patients with recurrent GBM.

**Methods:**

ARISTOCRAT is a phase II, multi-centre, double-blind, placebo-controlled, randomised trial to assess cannabinoids in patients with recurrent MGMT methylated GBM who are suitable for treatment with TMZ. Patients who have relapsed ≥ 3 months after completion of initial first-line treatment will be randomised 2:1 to receive either nabiximols or placebo in combination with TMZ. The primary outcome is overall survival time defined as the time in whole days from the date of randomisation to the date of death from any cause. Secondary outcomes include overall survival at 12 months, progression-free survival time, HRQoL (using patient reported outcomes from QLQ-C30, QLQ-BN20 and EQ-5D-5L questionnaires), and adverse events.

**Discussion:**

Patients with recurrent MGMT promoter methylated GBM represent a relatively good prognosis sub-group of patients with GBM. However, their median survival remains poor and, therefore, more effective treatments are needed. The phase II design of this trial was chosen, rather than phase III, due to the lack of data currently available on cannabinoid efficacy in this setting. A randomised, double-blind, placebo-controlled trial will ensure an unbiased robust evaluation of the treatment and will allow potential expansion of recruitment into a phase III trial should the emerging phase II results warrant this development.

**Trial registration:**

ISRCTN: 11460478. ClinicalTrials.Gov: NCT05629702.

**Supplementary Information:**

The online version contains supplementary material available at 10.1186/s12885-023-11792-4.

## Background

Glioblastoma (GBM) is the most common adult malignant brain tumour, with an incidence of 5 per 100,000 per year in England [1]. Prognosis is poor, with the majority of patients surviving less than one year and the average years of life lost to a brain tumour being 20.1, compared to breast cancer at 13.5 [2]. Treatment is palliative in nature from diagnosis, aimed at delaying disease progression and managing symptoms/preserving health-related quality of life (HRQoL). Optimal first-line treatment includes debulking surgery (if feasible), followed by chemo-radiotherapy and adjuvant chemotherapy using temozolomide (TMZ) (Stupp regimen) [3]. Despite this intensive treatment, median progression free survival time (PFS) is 6.9 months and median overall survival time (OS) 14.6 months [3]. At the time of inevitable disease recurrence, the aim remains broadly similar to first line treatment; delaying further progression and maintaining HRQoL. There is no agreed standard treatment, therefore, treatment decisions are made on a case-by-case basis [4].

Response to TMZ is an important determinant of outcome. Patients with O^6^-methylguanine-DNA-methyltransferase (MGMT) promoter methylated tumours have better outcomes from treatment with TMZ, compared to those with promoter unmethylated tumours [5, 6]. Patients with tumours showing MGMT promoter methylation represent around 40% of newly diagnosed GBM [7, 8]. Common practice includes a further course of TMZ in those patients with MGMT methylated tumours who relapse more than six months after completing adjuvant TMZ.

Phytocannabinoids occur naturally in cannabis plants and have been used medicinally for centuries for a variety of purposes [9]. Δ9-tetrahydrocannabinol (THC) is the major psychoactive constituent in cannabis, and cannabidiol (CBD) the major non-psychoactive constituent. Activation of the cannabinoid receptors, CB1 and CB2, exerts various downstream signalling effects, with diverse consequences on cellular biology and functions [10]. Glioblastomas express both CB1 and CB2 [11], with high-grade tumours expressing high levels of CB2. In vivo studies have found that the administration of CBD and THC reduced tumour growth in animal models of glioma [12]. These effects are thought to be mediated by induction of cell death (via apoptosis or cytotoxic autophagy), inhibition of cell proliferation, and anti-angiogenic effects [13]. Specific to GBM, combined administration of THC and TMZ exerts strong anti-tumoural effects in glioma xenografts, an effect maintained in tumours resistant to TMZ treatment [14]. In a trial investigating the effects of intracranial THC (100 mg/ml in ethanol) in patients with recurrent GBM, a reduction in tumour cell proliferation was reported in two of nine patients [15].

Jazz Pharmaceuticals (previously GW Pharma Ltd.) have developed nabiximols (trade name Sativex®), an oromucosal spray of a complex botanical mixture containing THC and CBD as the principal cannabinoids, with additional cannabinoid constituents and non-cannabinoid components, and was the first cannabis-based medicine to be licensed in the UK for multiple sclerosis spasticity. A two-part safety and exploratory efficacy randomised phase I double blind, placebo-controlled trial of nabiximols in combination with dose dense TMZ in 21 patients with relapsed GBM suggested that combination nabiximols/TMZ may be associated with prolonged OS compared to TMZ alone (observed hazard ratio < 0.5). In this trial, 12 patients received nabiximols and 9 placebo due to an imbalance in randomisation meaning that the interpretation of OS needed to be done with caution [16]. The most common adverse events (AEs) reported were vomiting, dizziness, nausea, fatigue, and headache, most commonly Common Terminology Criteria for Adverse Events (CTCAE) grade 2 and 3, and there was no difference in the incidence of treatment emergent AEs between the treatment groups [16]. There was no interaction documented between nabiximols and TMZ [16].

These data require confirmation in the setting of a larger randomised study in which known biomarkers of response to treatment, including MGMT status, are accounted for. Therefore, the ARISTOCRAT trial will assess whether the addition of cannabinoid (nabiximols) to standard TMZ treatment in patients with recurrent MGMT methylated GBM improves disease outcomes.

## Methods

### Trial design

**Fig. 1:**
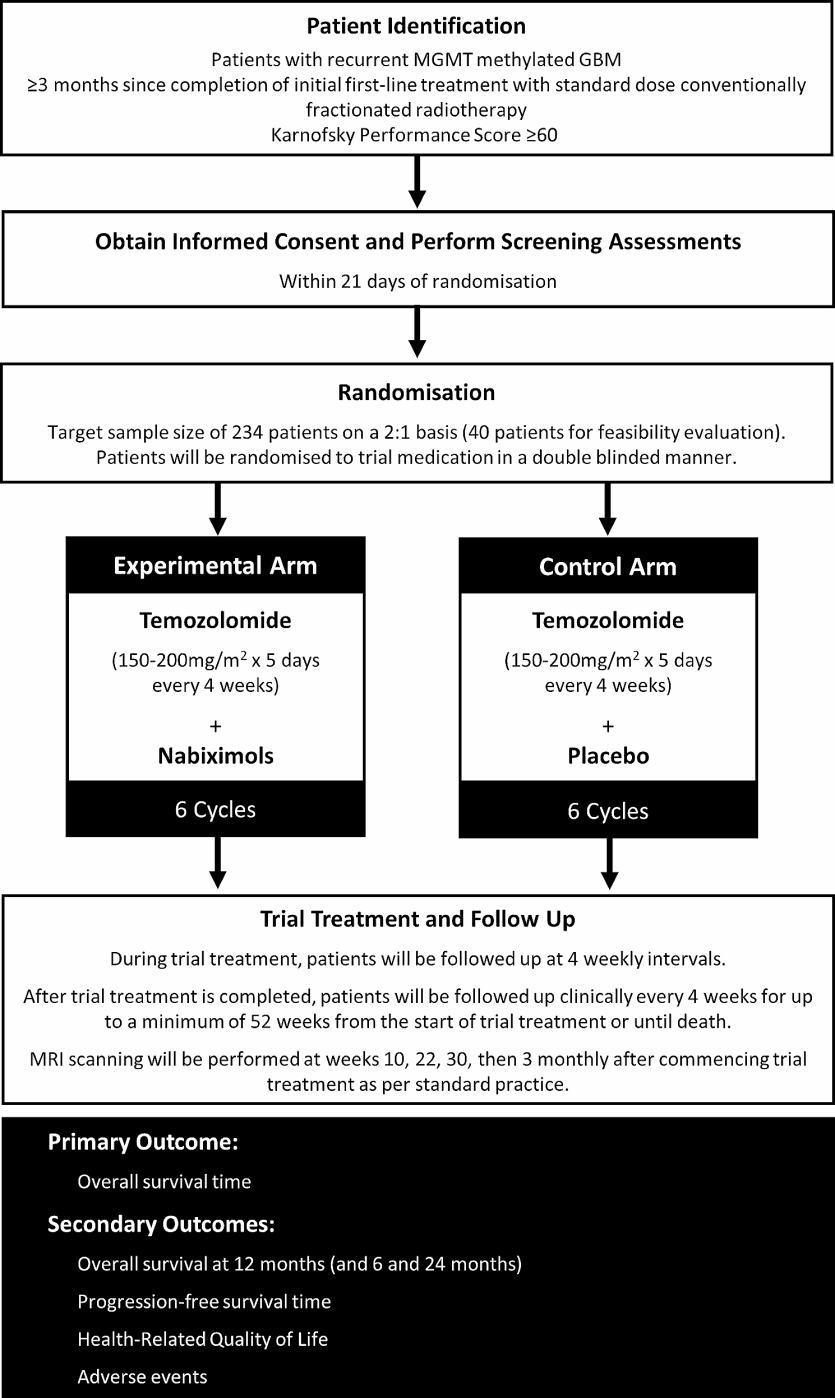
ARISTOCRAT trial schema Overview of ARISTOCRAT trial GBM: Glioblastoma; MGMT: O^6^-Methylguanine-DNA-methyltransferase

ARISTOCRAT is a phase II, multi-centre, double-blind, placebo-controlled, randomised trial to compare nabiximols with placebo in patients with recurrent MGMT methylated GBM suitable for treatment with TMZ. The trial has a target sample size of 234 patients who will be randomised on a 2:1 basis to receive either nabiximols or placebo, respectively, in combination with standard TMZ treatment (Fig. 1). The trial includes an initial feasibility study of 40 patients to confirm safety, compliance, and achievability of planned target recruitment. There are no formal criteria for evaluating feasibility but once 40 patients have been recruited, the independent Data Monitoring Committee (DMC) will review AE data, details on protocol treatment received, monthly recruitment rates and projected recruitment to make recommendations on trial continuation.

The phase II trial design will enable potential expansion of recruitment into a phase III trial, should the emerging phase II results warrant this. Should this occur, funding will be sought, and the protocol amended.

A list of the ARISTOCRAT trial sites can be requested from the ARISTOCRAT Trial Office (ARISTOCRAT@trials.bham.ac.uk). The Standard Protocol Items: Recommendations for Intervention Trials (SPIRIT) checklist [17] is provided in Appendix 1. The World Health Organization (WHO) Trial Registration Data Set is provided in Appendix 2.

### Randomisation and blinding

Randomisation is on a 2:1 basis to receive either nabiximols or placebo and stratified by age (≤ 70 vs. > 70 years), performance status (Karnofsky PS 60–79 v 80–100), and sex (male vs. female) in a double-blinded manner.

Treatment allocation is performed by central randomisation at the Cancer Research UK Clinical Trials Unit (CRCTU), University of Birmingham following submission of the completed Randomisation Form on the electronic Remote Data Capture (eRDC) system. A standard minimisation procedure is being used to allocate patients into the two arms to ensure a balance between the stratification variables.

Each patient will be assigned a unique Spray Vial Allocation Number, which will correspond to the Spray Vial Allocation List provided to local pharmacy at the site initiation. This list is strictly for pharmacy staff and will not be disclosed to any of the other members of the clinical team at the site to maintain blinding. The local pharmacy will then blind the appropriate medication for that patient. Neither the investigator nor the patient will know which combination is being administered.

Emergency unblinding will only be available in the event of patient safety concerns, this can be performed via the ARISTOCRAT Trial Office or via the site pharmacy using the eRDC system or paper allocation list.

### Patient and public involvement

Patient and public involvement and engagement (PPIE) has been integral to this study from its inception. The study was discussed by patients at The Brain Tumour Charity’s Research Involvement Network, and feedback received was used in developing the trial design. Our patient and public advisor, Peter Buckle, co-developed the ARISTOCRAT trial by reviewing and refining the protocol and the participant-facing documents. He has provided input into the patient-reported outcome measures and has guided messaging about the study for the community. As a member of the Trial Management Group (TMG), he continues to assess trial conduct and will contribute to the interpretation and dissemination of study findings. A second PPIE representative, Kathy Seddon, sits on the Trial Steering Committee (TSC).

A video interview was recorded between the Chief Investigator and a GBM patient as an educational tool for the trial and was posted online. In addition, a patient-facing website has been developed with our PPIE representatives and is hosted by the University of Birmingham (www.birmingham.ac.uk/aristocrat), along with lay-friendly materials posted to The Brain Tumour Charity’s website.

Our ongoing plans for dissemination include a webinar hosted by The Brain Tumour Charity to be held following the launch of the trial to update patients, the public, and supporters.

### Patient recruitment

This trial aims to recruit patients at UK participating centres following referral from their local multidisciplinary team at point of recurrence, including confirmation of radiological recurrence from a consultant neuro-radiologist. Recruitment will be over three years, with participants followed up for at least one year from the start of treatment or until death. Key eligibility criteria are listed in Table 1.

**Table 1:** Key eligibility criteria for ARISTOCRAT

**Inclusion criteria**
Age ≥ 16
Karnofsky Performance Status ≥ 60
Histological diagnosis of MGMT promoter methylated, IDH wild type GBM with consistent local molecular pathology
First recurrence of GBM planned for systemic treatment are most in keeping with recurrence and not pseudo-progression and patient is planned for systemic treatment
Patients must have received initial first-line treatment with standard dose conventionally fractionated radiotherapy (i.e., 40 Gy in 15 fractions or 54-54-60 Gy in 28–33 fractions; other regimes may be considered in consultation with the ARISTOCRAT Trial Office) with concomitant and adjuvant TMZ
• A minimum of 3 cycles of adjuvant TMZ must have been received
• A minimum of SD (or PR/CR) at the end of first-line treatment
≥ 3 months since day 28 of the last cycle of TMZ
Adequate hematologic, renal, and hepatic function within 14 days prior to randomisation
**Exclusion criteria**
Pathology inconsistent with IDH wild type GBM
Prior invasive malignancy (except non-melanoma skin cancer), unless disease free for a minimum of one year
Prior treatment with stereotactic radiotherapy, brachytherapy, or convection enhanced delivery of any agent
Prior treatment, apart from debulking surgery, for first recurrence of GBM
Personal history of schizophrenia, other psychotic illness, severe personality disorder or other significant psychiatric diagnosis other than depression associated with their underlying glioma condition
Prior allergic reaction or significant toxicity (≥ Grade 3 CTCAE) related to TMZ treatment
Current or recent cannabis or cannabinoid-based medications within 30 days of randomisation and/or unwilling to abstain for the duration of the trial
Women who are pregnant, breastfeeding or a woman of childbearing potential who is unwilling to use effective contraceptive methods during trial treatment and for 6 months after completion of trial treatment
• Women of childbearing age must have a negative pregnancy test within 7 days prior to randomisation
Men who are sexually active and unwilling/unable to use medically acceptable forms of contraception during trial treatment or for 6 months after completion of trial treatment
Contra-indication to MRI or gadolinium
Hereditary galactose intolerance, total lactase deficiency or glucose-galactose malabsorption
Known history of current or prior alcohol or drug dependence
Unable to administer oromucosal medication due to mucosal lesions or other issues

CR: complete response; CTCAE: Common Terminology Criteria for Adverse Events; GBM: glioblastoma; IDH: isocitrate dehydrogenase; MGMT: O^6^-methylguanine-DNA-methyltransferase; MRI: magnetic resonance imaging; PR: partial response; SD: stable disease; TMZ: temozolomide

ARISTOCRAT is linked to the Tessa Jowell BRAIN MATRIX (TJBM) programme; utilising the TJBM infrastructure, opening the same participating sites, and aligning the data collection and quality of life assessments already embedded in TJBM [18]. Therefore, patients registered within the TJBM platform who are potentially eligible for ARISTOCRAT may be identified and suggested to sites for consideration in the trial.

### Screening and consent

Written informed consent will be obtained from all patients. The investigator will provide information to allow the patient to make an informed decision regarding their participation. The patient summary and information sheet are provided in Appendix 3, with an exemplar informed consent form in Appendix 4. If a participant loses mental capacity during the study, their responsible caregiver will be consulted to determine their suitability of remaining on trial. Any data collected up to the point of withdrawal will be kept on record and used in the trial analysis. If the patient loses capacity and the caregiver decides that the patient may continue to participate in the trial, then the caregiver will be asked to provide trial related information (e.g., information on side effects) on behalf of the patient.

### Interventions

Patients will be randomised to one of the two treatment arms:

- Experimental arm: TMZ with nabiximols.
- Control arm: TMZ with nabiximols-matched placebo.

All patients will receive non-dose dense TMZ given at 4-weekly intervals (± 3 days for unavoidable delays, bank holidays etc.) up to a maximum of six cycles (pending tolerability) or until disease progression, whichever is sooner. It is recommended that initial doses (in the absence of renal or hepatic impairment) will be:

- Cycle 1: TMZ 150mg/m^2^, with dose capping as per local standard practice, once daily (OD) for days 1–5, by mouth (PO), on a 28-day cycle for cycle 1.
- Cycles 2–6: TMZ 200mg/m^2^, with dose capping as per local standard practice, OD for days 1–5, PO, on a 28-day cycle for cycles 2–6.

Patients who were unable to tolerate 200mg/m^2^ during their initial adjuvant treatment, are permitted to start trial treatment on a lower dose of 150 mg/m^2^ with dose capping as per local standard practice.

Nabiximols or placebo is administered in a self-titration regime (Table 2). This should start as a single spray in the evening of day 1 cycle 1 and gradually titrate to reach an individualised dose based on tolerability. A minimum of three sprays per day is recommended, with the maximum daily dose of 12 sprays. Patients will establish their individualised maximum tolerated dose (MTD) within 14 days of their first dose and continue this regime every day for up to six months (six cycles). If this is not achievable the patient should be permitted to continue in the trial, providing that they continue to receive trial medication, but will not be considered evaluable for the trial and will be replaced.

**Table 2:** Nabiximols/placebo titration regimen

Day	Number of Sprays		
	Morning Between waking-up and 12pm	Afternoon Between 4pm and bedtime	Total number of sprays per day
1	0	1	1
2	0	1	1
3	0	2	2
4	0	2	2
5	1	2	3
6	1	3	4
7	1	4	5
8	2	4	6
9	2	5	7
10	3	5	8
11	3	6	9
12	4	6	10
13	4	7	11
14	5	7	12

Taken from the nabiximols Summary of Product Characteristics [29]

This trial uses a non-commercial supply of nabiximols outside its licenced indication, and is a Class B controlled drug under the Misuse of Drugs Act 1971 so is placed in Schedule 2 of the Misuse of Drug Regulations 2001. All trial sites are therefore required to hold an appropriate Home Office licence, or exemption, and to follow Schedule 2 requirements for the storage and dispensing of nabiximols, adding complexity to the pharmacy aspects of this blinded trial.

Patients will be followed up at 4-weekly assessments for a minimum of 52 weeks from the start of trial treatment or until death, whichever is sooner. MRI scanning will be performed at screening, week 10, week 22, week 30, then 3-monthly after commencing trial treatment as per standard practice. A schedule of events is included in Table 3.

**Table 3:** ARISTOCRAT trial schedule of events

Activity	Screening Within 21 days of trial entry	Randomisation	Week 0^#^ Cycle 1	Week 4 Cycle 2 ± 3 days	Week 8 Cycle 3 ± 3 days	Week 10 ± 3 days	Week 12 Cycle 4 ± 3 days	Week 16 Cycle 5 ± 3 days	Week 20 Cycle 6 ± 3 days	Week 22 ± 3 days	Week 24 EOT	Follow Up		
												Week 28	Week 30	As per SoC
Informed consent^1^	X													
Confirm eligibility	X													
MRI scan (RANO criteria)^2^	X					X				X			X	X Minimum 3 monthly
Blood tests^3^	X		X	X	X		X	X	X		X			
Concomitant medication^4^	X		X	X	X		X	X	X		X	X		X
Electrocardiogram^5^	X													
Karnofsky Performance Status^6^	X		X	X	X		X	X	X		X	X		X
Medical history^7^	X													
Physical examination^8^	X		X	X	X		X	X	X		X			
Pregnancy test^9^	X		X	X	X		X	X	X		X			
Urine test for cannabinoid use^10^			X		X						X			
Vital signs^11^	X		X	X	X		X	X	X		X			
TMZ + nabiximols/placebo^12^			X	X	X		X	X	X					
Adverse Events^13^	X		X	X	X		X	X	X		X	X		X
Patient diary			X	X	X		X	X	X					
Health-Related Quality of Life^14^			X		X			X			X			
Survival status^14^			X	X	X		X	X	X		X	X		X

# Patients must start trial treatment within 28 days of randomisation

^1^ Written informed consent must be obtained before any trial procedures occur and no greater than 21 days of before randomisation

^2^ Brain MRI scan is ideally performed within 21 days of randomisation. If MRI scan has not been performed within 28 days of start of cycle 1, this will need to be repeated. During trial treatment and follow up, scans must be performed ± 3 days of the scheduled visit

^3^ Blood tests to include: Full blood count (to include Absolute Neutrophil Count and platelets count), urea and electrolytes, liver function tests. During trial treatment, should be performed within 14 days prior to Cycle 1 and within 3 days of Cycles 2–6

^4^ Review of concomitant medication to include dexamethasone and anti-epileptic use

^5^ Single echocardiogram to be performed at Screening only

^6^ Karnofsky Performance Status to be used

^7^ Pseudonymised copy of local pathology report for first diagnosis of GBM must be sent to the ARISTOCRAT Trial Office

^8^ Physical examination to include central and peripheral nervous system examination, required at baseline and subsequently if clinically indicated

^9^ Females of childbearing potential will require a negative pregnancy test (serum or urine) within 7 days prior to randomisationand within 3 days of Cycles 1-6 during treatment.

^10^ Urine sample for cannabinoid use on week 0 (Cycle 1) to be collected pre-dose

^11^ Vital signs to include blood pressure, heart rate, height (screening only) and weight

^12^ Temozolomide (TMZ): 150mg/^2^ for Cycle 1, increasing to 200mg/m^2^ for Cycles 2–6, once daily for days 1–5 at the start of each 28-day cycle

^13^ Adverse Events (AEs) to be documented and reported until the end of treatment visit. During follow up, AEs to be reviewed by Investigator but do not need to be recorded on the AE Form

^14^ HRQoL questionnaires to be completed in clinic at defined visits. HRQoL questionnaires on Week 0 (Cycle 1) to be completed pre-dose

^15^ Follow up visits and assessment will continue until a minimum of 52 weeks from the start of trial treatment or until death, whichever is sooner, as part of standard of care visits. To include documentation of additional hospital or GP visits at follow up visits

Although no additional translational samples will be collected during ARISTOCRAT, patients will provide additional consent for the collection of samples, including archival formalin-fixed paraffin-embedded tumour blocks, to allow for future translational research.

### Treatment compliance

Patients will be asked to complete patient diaries during each cycle of treatment to record the number of TMZ tablets taken and the number of sprays of nabiximols/placebo administered, as well as any side effects experienced. Missed doses will be recorded with reasons. Patients will be asked to return their diaries at their next clinic visit. Urine samples will be collected to check for ‘recreational’ cannabinoid (THC) testing at baseline, cycle three, and at end of treatment. This will be performed by Matrix Diagnostics Ltd. to maintain blinding; positive dipstick tests will receive confirmatory mass spectrometry analysis. Positive tests will not be fed back to sites or patients and will not affect a patient’s continued participation in the trial; however, they may be considered unevaluable at analysis.

### Dose modifications

TMZ dose modifications, delays, or discontinuation should be made in response to toxicities according to the institution’s usual practice. Amended doses and reasons for any modifications will be recorded at each cycle. Suggested guidance for modifications is provided in Appendix 5. If TMZ cycles are delayed due to TMZ related toxicities, then nabiximols/placebo can be continued during the delay if deemed appropriate by the treating clinician. Any patient with two prior dose reductions who experiences toxicity that would require a third dose reduction of TMZ or a delay of > 2 weeks will have their TMZ, and nabiximols/placebo discontinued and be withdrawn from the trial.

If a toxicity suspected to be related to nabiximols/placebo occurs, then dosing can be suspended or amended until the event has resolved. Patients, whose dose is adjusted, may restart the titration at an appropriate dose and return to their MTD gradually by increasing the number of sprays by one spray per day (Table 2). If a participant requires > 2 weeks off nabiximols/placebo or reduce their dose to < 3 sprays/day, then the patient should stop nabiximols/placebo permanently. TMZ can be continued, if appropriate, at the discretion of the treating physician as per local standard of care.

### Concomitant medication

Cannabis or cannabinoid-based medications (up to and including within 30 days of randomisation) are prohibited during the trial. Live vaccines should be avoided during and for six weeks after completion of chemotherapy. Patients are permitted to receive COVID-19 vaccines whilst on trial treatment.

Patients with genetic glucuronidation disorders (e.g., Gilbert’s disease) must be treated with caution when nabiximols is co-administered.

A review of patients’ dosing regimen is advised when co-administering nabiximols with CYP3A4 substrates and other drugs metabolised through cytochrome P-450 enzymes e.g., coumarins, statins, beta-blockers, and corticosteroids.

Concomitant treatment of nabiximols with strong enzyme inducers (e.g., rifampicin, carbamazepine, phenytoin, phenobarbital, St John’s Wort) should be avoided whenever possible.

Due to possible adverse interactions with nabiximols, sedatives, muscle relaxers, anti-spasticity agents, and alcohol these should be avoided.

### Outcome measures

The primary outcome is OS defined as the time in whole days from the date of randomisation to the date of death from any cause. Patients who are alive at the time of analysis will be censored at the date last seen alive. Secondary outcome measures are overall survival at 12 months (OS12) (of particular clinical relevance is the overall survival at 12 months from the date of randomisation with overall survival at six months and 24 months also of interest); PFS, defined as the time in whole days from date of randomisation to the date of the first documented evidence of disease progression or death (from any cause); and HRQoL assessed at screening, and then every eight weeks until the end of treatment. HRQoL will be assessed with the EORTC QLQ-C30 [19, 20], EORTC BN20 [21, 22], single items from the EORTC item library, and the EQ-5D-5L [23].

### Statistical analysis plan

#### Sample size

The target sample size is 234 patients based on testing the null hypothesis of no difference between the treatment arms in terms of OS, i.e., a hazard ratio (HR) = 1 and assumes exponential distribution for survival times. Calculations assume a recruitment period of 18 months and follow-up period of 12 months. As a phase II trial, the design uses a relaxed one-sided significance level of 10% (i.e., α = 0.1) and a randomisation ratio of 2:1 in favour of the experimental treatment arm. The expected median survival time on the placebo arm is 12 months, which equates to a hazard rate of 0.058 [6, 24]. Given these design parameters, the trial requires 132 events to have 80% power (i.e., β = 0.2) to detect a difference with a hazard ratio of 0.68, i.e., median survival time of 17.6 months on the experimental treatment arm and hazard rate of 0.039. Given the results from the phase I trial [16], we believe this is a plausible benefit to target. It is estimated that we need to recruit 222 patients during the 18-month recruitment period to observe this number of events; a further 5% to cover potential dropouts has been added to give a target sample size of 234. The trial includes an initial feasibility study that, based on a pragmatic rationale, will incorporate the first 40 patients recruited.

#### Interim analysis

An interim analysis will occur at the end of the feasibility study with 40 patients and every six months thereafter and results reported to the DMC. Interim data from the feasibility study (numbers of participating centres and monthly recruitment rates) will also be presented to the DMC to evaluate recruitment feasibility of the 234 target. In addition, treatment compliance, AE incidence, and HRQoL data will be reviewed to evaluate if the intervention is safe and acceptable. Later interim analyses will focus on efficacy outcome measures as well as safety. In particular, the DMC will advise on whether the emerging data warrant the potential expansion into a phase III trial. Should the DMC make this recommendation then further funding would be required.

#### Outcome analyses

Overall survival time will be analysed using the Kaplan-Meier method to estimate and plot the survivor functions for each treatment arm, together with summary statistics of median and 12-month survival rates. In addition, 6-month and where available 24-month survival rates will be reported. The difference between treatment arms will be estimated using a hazard ratio generated from a Cox regression model. All estimates will be reported with one-sided 90% confidence intervals. The null hypothesis of no difference between the treatment arms in terms of overall survival time will be tested using the Cox regression model. The primary analysis will be on all patients who have received at least two cycles of protocol treatment.

Progression-free survival time will be analysed in the same way as OS. Measures of HRQoL will be reported on each treatment arm as means over time with 95% confidence intervals. The incidence of adverse events will be reported in the case report form (CRF) and patient diaries on each arm and compared descriptively.

Final analysis of the completed phase II trial will occur once all participants have been recruited and followed up for at least 12 months from closure of the trial to recruitment.

The treatment effect will be evaluated for subgroups of patients defined by age (≤ 70 vs. > 70 years), performance status (Karnofsky Performance Status 60–79 vs. 80–100), sex (male vs. female), symptomatic vs. asymptomatic at baseline, numbers of sprays per day and re-resection group.

#### Adverse event reporting

Collection and reporting of AEs will be as per CTCAE, version v5.0 [25]. Definitions of different AE types are listed in Appendix 6. The investigator should assess seriousness and causality (relatedness) of all AEs experienced by the patient and documented in the source data, with reference to the Summary of Product Characteristics (SmPC). All medical occurrences, which meet the definition of an AE should be reported but includes abnormal laboratory findings of grade 3 and above only. The following events should not be reported as serious adverse events (SAEs):

- Hospitalisations for:
  - Protocol defined treatment.
  - Pre-planned elective procedures unless the condition worsens.
  - Treatment for progression of the patient’s cancer.
- Progression or death as a result of the patient’s cancer, as this information is captured elsewhere on the CRF.

Details of all AEs (except those listed above) will be documented and reported from the date of commencement of protocol defined treatment until 30 days after the administration of the last treatment.

### Data management and monitoring

The CRF will be completed via an eRDC system, access to which will be granted by the ARISTOCRAT Trial Office. SAE reporting will be paper based. Data reported on each form should be consistent with the source data or the discrepancies explained. If information is not known, this must be indicated. All missing and ambiguous data will be queried. All sections are to be completed before returning.

All trial records must be archived and securely retained for at least 25 years after the end of the trial. On-site monitoring will be carried out as required following a risk assessment and as documented in the Quality Management Plan. Monitoring activities will be reported to the central ARISTOCRAT Trial Office and any issues noted will be followed up to resolution. ARISTOCRAT will also be centrally monitored, which may trigger additional on-site monitoring.

The CRCTU will hold the final trial dataset and will be responsible for the controlled sharing of anonymised clinical trial data with the wider research community to maximise potential patient benefit while protecting the privacy and confidentiality of trial participants. Data anonymised in compliance with the Information Commissioner’s Office requirements, using a procedure based on guidelines from the Medical Research Council Methodology Hubs [26] will be available for sharing with researchers outside of the trials team within 12 months of the primary publication.

### Trial organisational structure

The sponsor is University of Birmingham: Research Governance & Integrity, Research Strategy and Services Division, Research Park, Birmingham, B15 2TT. Email: researchgovernance@contacts.bham.ac.uk. The trial is being conducted under the auspices of the CRCTU, University of Birmingham according to their local procedures. The TMG will comprise the Chief Investigators, Co-investigators, Trial Management Team Leader, Senior Trial Coordinator, Trial Coordinator, Lead and Trial Statistician, Trial Monitor, and patient representatives. The TMG will be responsible for the day-to-day conduct of the trial and will meet at regular intervals (e.g., every three months), or as required, usually by teleconference.

The TSC will provide oversight of the trial on behalf of the Sponsor (University of Birmingham) and trial funder (The Brain Tumour Charity) to ensure that the trial conduct, concentrating on progress, adherence to the protocol, patient safety, and consideration of new information of relevance to the research question(s). The TSC will provide advice, through its chair, to the Chief Investigator and the University of Birmingham on all appropriate aspects of the trial. The TSC will be asked to comment in detail on substantial changes to the protocol. The TSC will meet at least once per year during recruitment.

The DMC will consist of at least two independent clinicians, as well as an independent statistician. Data analyses will be supplied in confidence to them, and advice garnered on whether the accumulated data from the trial, together with the results from other relevant research, justifies the continuing recruitment of further patients. The DMC will operate to a trial specific charter based upon the template created by the Damocles Group. During the recruitment phase of the trial, the DMC will review the unblinded safety and efficacy data in accordance with the guidelines for the pre-planned interim analysis. The DMC will meet every six months during trial recruitment. Additional meetings may be called if recruitment is much faster than anticipated and the DMC may, at their discretion, request to meet more frequently or continue to meet following completion of recruitment. An emergency meeting may also be convened if a safety issue is identified.

The DMC will report directly to the TMG who will convey the findings of the DMC to TSC. The DMC may consider recommending the discontinuation of the trial if the recruitment rate or data quality are unacceptable or if any issues are identified that may compromise patient safety. The trial may also stop early if the interim analyses showed a difference between treatments that were deemed to be convincing to the clinical community.

### Confidentiality and data protection

Personal data recorded on all documents will be regarded as strictly confidential and will be handled, stored, and processed in accordance with the GDPR and the Data Protection Act 2018. As specified in the patient information sheet and with the patients’ consent, patients will be identified using only their unique trial number, initials, and date of birth. Authorised staff may have access to the records for quality assurance and audit purposes. The Trials Office maintains the confidentiality of all patients’ data and will not disclose information by which patients may be identified to any third party other than those directly involved in the treatment of the patient and organisations for which the patient has given explicit consent for data transfer (e.g., laboratory staff).

### Dissemination of results and publication policy

Results of the primary and secondary endpoints will be submitted for publication in peer-reviewed journals. Manuscripts will be prepared by the TMG and authorship determined by mutual agreement. A lay summary of the results will be published online and provided to trial sites to share with patients.

### Trial status

Recruitment for the trial opened on 03-Feb-2023 and is expected to last three years.

## Discussion

### Choice of trial design

A phase II rather than phase III trial design has been chosen for the ARISTOCRAT trial, as the preliminary data on potential efficacy is still sparse. In addition, the feasibility of undertaking such a trial in terms of safety, compliance, and achievability of recruitment needs to be evaluated before embarking on a phase III and this will be done within the early stages of this trial. The choice for the design to be a randomised, double-blind, placebo-controlled trial will ensure an unbiased robust evaluation of the treatment and will allow potential recruitment expansion into a phase III trial should the emerging phase II results warrant this development. In this case, the phase II sample size is synergistic with the requirements for the first stage of a two-arm two-stage phase III trial design.

### Choice of trial treatment regimens

Within ARISTOCRAT, all patients will be treated with non-dose dense (150-200mg/m^2^ 5d/28d) TMZ. This was justified due to subsequent studies having not established the superiority of dose-dense TMZ compared to monthly dosing since completion of the phase I study [27, 28]. Furthermore, due to the substantial between-patient variation in pharmacokinetics and pharmacodynamics of cannabinoids, an individualised dose paradigm is being used in this trial as was used in the previous phase I study.

### Trial delivery

Patients on the control arm of ARISTOCRAT will have received non-dose dense TMZ with nabiximols-matched placebo and, therefore, the data collected on this arm may be used as a backbone for future trials in accordance with the TJBM study. This collaboration will allow data sharing across the two studies thereby streamlining patient entry and provide additional oversight of ARISTOCRAT through the TJBM programme. In addition, patients also participating in TJBM will provide paired tumour and blood samples at the time of entry to TJBM, which are used for molecular analysis and whole genome sequencing [18]. Any excess sample will be stored at the TJBM Tissue Bank and available for future ethically approved research studies. Samples are also collected at any other point of biopsy or resection. For these patients, consent will be taken for non-anonymised samples, pathology reports and imaging required for accurate pathology review and quality assurance purposes to be transferred to the pathology team using NHS pathways.

Patients are also encouraged to participate in The Brain Tumour Charity’s BRIAN (Brain tumouR Information and Analysis Network) study (REC Reference: 18/SC/0283, IRAS No.: 237931), which allows them to record their experiences of having a brain tumour using a mobile phone or web app. ARISTOCRAT trial data will also be linked and shared with the BRIAN database to better understand the effects of a brain tumour on patient HRQoL.

### Electronic supplementary material

Below is the link to the electronic supplementary material.

Supplementary Material 1

## Data Availability

Not applicable.

## Abbreviations

AE: Adverse event
ANC: Absolute Neutrophil Count
AR: Adverse reaction
BRIAN: Brain tumouR Information and Analysis Network
CBD: Cannabidiol
CRCTU: Cancer Research UK Clinical Trials Unit
CRF: Case Report Form
CTCAE: Common Terminology Criteria for Adverse Events
DMC: Data Monitoring Committee
eRDC: Electronic Remote Data Capture
GBM: Glioblastoma
HRQoL: Health-Related Quality of Life
MGMT: O^6^-Methylguanine-DNA-methyltransferase
OD: Once daily
OS: Overall survival time
OS12: 12 month overall survival
PFS: Progression-free survival time
PO: By mouth
PPIE: Patient public involvement and engagement
SAE: Serious adverse event
SAR: Serious adverse reaction
SmPC: Summary of Product Characteristics
SUSAR: Suspected Unexpected Serious Adverse Reaction
THC: Tetrahydrocannabinol
TJBM: Tessa Jowell BRAIN MATRIX
TMG: Trial management group
TSC: Trial steering committee
TMZ: Temozolomide
