# Supplementary material for: A randomised phase II trial of temozolomide with or without cannabinoids in patients with recurrent glioblastoma (ARISTOCRAT): protocol for a multi-centre, double-blind, placebo-controlled trial
Source: BMC Cancer. 2024 Jan 15;24:83. doi: 10.1186/s12885-023-11792-4 (PMC10790538; doi:10.1186/s12885-023-11792-4)
Supplement: Supplementary file 1 — Supplementary Material 1 [file 12885_2023_11792_MOESM1_ESM.pdf]

Appendix 1: ARISTOCRAT SPIRIT checklist

Reporting checklist for protocol of a clinical trial.

Based on the SPIRIT guidelines.

Instructions to authors

Complete this checklist by entering the page numbers from your manuscript where readers will find each of the items listed below.

Your article may not currently address all the items on the checklist. Please modify your text to include the missing information. If you are certain that an item does not apply, please write "n/a" and provide a short explanation.

Upload your completed checklist as an extra file when you submit to a journal.

In your methods section, say that you used the SPIRITreporting guidelines, and cite them as:

Chan A-W, Tetzlaff JM, Gøtzsche PC, Altman DG, Mann H, Berlin J, Dickersin K, Hróbjartsson A, Schulz KF, Parulekar WR, Krleža-Jerić K, Laupacis A, Moher D. SPIRIT 2013 Explanation and Elaboration: Guidance for protocols of clinical trials. BMJ. 2013;346:e7586

		Reporting Item	Page Number
**Administrative information**			
Title	#1	Descriptive title identifying the study design, population, interventions, and, if applicable, trial acronym	1
Trial registration	#2a	Trial identifier and registry name. If not yet registered, name of intended registry	3
Trial registration: data set	#2b	All items from the World Health Organization Trial Registration Data Set	Appendix 2
Protocol version	#3	Date and version identifier	22
Funding	#4	Sources and types of financial, material, and other support	22 + 23
Roles and responsibilities: contributorship	#5a	Names, affiliations, and roles of protocol contributors	1 + 23

Roles and responsibilities: sponsor contact information	#5b	Name and contact information for the trial sponsor	16
Roles and responsibilities: sponsor and funder	#5c	Role of study sponsor and funders, if any, in study design; collection, management, analysis, and interpretation of data; writing of the report; and the decision to submit the report for publication, including whether they will have ultimate authority over any of these activities	23
Roles and responsibilities: committees	#5d	Composition, roles, and responsibilities of the coordinating centre, steering committee, endpoint adjudication committee, data management team, and other individuals or groups overseeing the trial, if applicable (see Item 21a for data monitoring committee)	16-18
**Introduction**			
Background and rationale	#6a	Description of research question and justification for undertaking the trial, including summary of relevant studies (published and unpublished) examining benefits and harms for each intervention	3-6
Background and rationale: choice of comparators	#6b	Explanation for choice of comparators	9-10
Objectives	#7	Specific objectives or hypotheses	5-6
Trial design	#8	Description of trial design including type of trial (eg, parallel group, crossover, factorial, single group), allocation ratio, and framework (eg, superiority, equivalence, non-inferiority, exploratory)	6
**Methods: Participants, interventions, and outcomes**			

Study setting	#9	Description of study settings (eg, community clinic, academic hospital) and list of countries where data will be collected. Reference to where list of study sites can be obtained	6 + 8
Eligibility criteria	#10	Inclusion and exclusion criteria for participants. If applicable, eligibility criteria for study centres and individuals who will perform the interventions (eg, surgeons, psychotherapists)	8<br><br>Table 1
Interventions: description	#11a	Interventions for each group with sufficient detail to allow replication, including how and when they will be administered	9 + 10
Interventions: modifications	#11b	Criteria for discontinuing or modifying allocated interventions for a given trial participant (eg, drug dose change in response to harms, participant request, or improving / worsening disease)	11 + 12; Table 2; Appendix 5
Interventions: adherance	#11c	Strategies to improve adherence to intervention protocols, and any procedures for monitoring adherence (eg, drug tablet return; laboratory tests)	11
Interventions: concomitant care	#11d	Relevant concomitant care and interventions that are permitted or prohibited during the trial	12
Outcomes	#12	Primary, secondary, and other outcomes, including the specific measurement variable (eg, systolic blood pressure), analysis metric (eg, change from baseline, final value, time to event), method of aggregation (eg, median, proportion), and time point for each outcome. Explanation of the clinical relevance of chosen efficacy and harm outcomes is strongly recommended	12-13
Participant timeline	#13	Time schedule of enrolment, interventions (including any run-ins and washouts), assessments, and visits for participants. A schematic diagram is highly recommended (see Figure)	Table 3
Sample size	#14	Estimated number of participants needed to achieve study objectives and how it was determined,	13

		including clinical and statistical assumptions supporting any sample size calculations	
Recruitment	#15	Strategies for achieving adequate participant enrolment to reach target sample size	n/a
**Methods: Assignment of interventions (for controlled trials)**			
Allocation: sequence generation	#16a	Method of generating the allocation sequence (eg, computer-generated random numbers), and list of any factors for stratification. To reduce predictability of a random sequence, details of any planned restriction (eg, blocking) should be provided in a separate document that is unavailable to those who enrol participants or assign interventions	7
Allocation concealment mechanism	#16b	Mechanism of implementing the allocation sequence (eg, central telephone; sequentially numbered, opaque, sealed envelopes), describing any steps to conceal the sequence until interventions are assigned	7
Allocation: implementation	#16c	Who will generate the allocation sequence, who will enrol participants, and who will assign participants to interventions	7
Blinding (masking)	#17a	Who will be blinded after assignment to interventions (eg, trial participants, care providers, outcome assessors, data analysts), and how	7
Blinding (masking): emergency unblinding	#17b	If blinded, circumstances under which unblinding is permissible, and procedure for revealing a participant's allocated intervention during the trial	7
**Methods: Data collection, management, and analysis**			
Data collection plan	#18a	Plans for assessment and collection of outcome, baseline, and other trial data, including any related	16

		processes to promote data quality (eg, duplicate measurements, training of assessors) and a description of study instruments (eg, questionnaires, laboratory tests) along with their reliability and validity, if known. Reference to where data collection forms can be found, if not in the protocol	
Data collection plan: retention	#18b	Plans to promote participant retention and complete follow-up, including list of any outcome data to be collected for participants who discontinue or deviate from intervention protocols	n/a
Data management	#19	Plans for data entry, coding, security, and storage, including any related processes to promote data quality (eg, double data entry; range checks for data values). Reference to where details of data management procedures can be found, if not in the protocol	16
Statistics: outcomes	#20a	Statistical methods for analysing primary and secondary outcomes. Reference to where other details of the statistical analysis plan can be found, if not in the protocol	14-15
Statistics: additional analyses	#20b	Methods for any additional analyses (eg, subgroup and adjusted analyses)	15
Statistics: analysis population and missing data	#20c	Definition of analysis population relating to protocol non-adherence (eg, as randomised analysis), and any statistical methods to handle missing data (eg, multiple imputation)	14
**Methods: Monitoring**			
Data monitoring: formal committee	#21a	Composition of data monitoring committee (DMC); summary of its role and reporting structure; statement of whether it is independent from the sponsor and competing interests; and reference to where further details about its charter can be found, if not in the protocol. Alternatively, an explanation of why a DMC is not needed	17

Data monitoring: interim analysis	#21b	Description of any interim analyses and stopping guidelines, including who will have access to these interim results and make the final decision to terminate the trial	14
Harms	#22	Plans for collecting, assessing, reporting, and managing solicited and spontaneously reported adverse events and other unintended effects of trial interventions or trial conduct	15
Auditing	#23	Frequency and procedures for auditing trial conduct, if any, and whether the process will be independent from investigators and the sponsor	16
**Ethics and dissemination**			
Research ethics approval	#24	Plans for seeking research ethics committee / institutional review board (REC / IRB) approval	21-22
Protocol amendments	#25	Plans for communicating important protocol modifications (eg, changes to eligibility criteria, outcomes, analyses) to relevant parties (eg, investigators, REC / IRBs, trial participants, trial registries, journals, regulators)	22
Consent or assent	#26a	Who will obtain informed consent or assent from potential trial participants or authorised surrogates, and how (see Item 32)	8
Consent or assent: ancillary studies	#26b	Additional consent provisions for collection and use of participant data and biological specimens in ancillary studies, if applicable	10
Confidentiality	#27	How personal information about potential and enrolled participants will be collected, shared, and maintained in order to protect confidentiality before, during, and after the trial	18
Declaration of interests	#28	Financial and other competing interests for principal investigators for the overall trial and each study site	22

Data access	#29	Statement of who will have access to the final trial dataset, and disclosure of contractual agreements that limit such access for investigators	16
Ancillary and post trial care	#30	Provisions, if any, for ancillary and post-trial care, and for compensation to those who suffer harm from trial participation	n/a
Dissemination policy: trial results	#31a	Plans for investigators and sponsor to communicate trial results to participants, healthcare professionals, the public, and other relevant groups (eg, via publication, reporting in results databases, or other data sharing arrangements), including any publication restrictions	18
Dissemination policy: authorship	#31b	Authorship eligibility guidelines and any intended use of professional writers	18
Dissemination policy: reproducible research	#31c	Plans, if any, for granting public access to the full protocol, participant-level dataset, and statistical code	n/a
**Appendices**			
Informed consent materials	#32	Model consent form and other related documentation given to participants and authorised surrogates	Appendices 3 + 4
Biological specimens	#33	Plans for collection, laboratory evaluation, and storage of biological specimens for genetic or molecular analysis in the current trial and for future use in ancillary studies, if applicable	n/a

Note: The SPIRIT Explanation and Elaboration paper is distributed under the terms of the Creative Commons Attribution License CC-BY-NC. This checklist can be completed online using https://www.goodreports.org/, a tool made by the EQUATOR Network in collaboration with Penelope.ai

Appendix 2: The WHO Trial Registration Data Set for ARISTOCRAT

Data category	Information
Primary registry and trial identifying number	ISRCTN11460478
Date of registration in primary registry	25-Oct-2022
Secondary identifying numbers	Clinical Trials.Gov NCT05629702
Source(s) of monetary or material support	Trial funding: The Brain Tumour Charity<br>Nabiximols and matched placebo provided free of charge by Jazz Pharmaceuticals.
Primary sponsor	University of Birmingham
Secondary sponsor(s)	n/a
Contact for public queries	aristocrat@trials.bham.ac.uk
Contact for scientific queries	aristocrat@trials.bham.ac.uk
Public title	A trial looking at Sativex for people with glioblastoma (ARISTOCRAT)

Data category	Information
Scientific title	ARISTOCRAT: A randomised controlled phase II trial of temozolomide with or without cannabinoids in patients with recurrent glioblastoma
Countries of recruitment	UK
Health condition(s) or problem(s) studied	Recurrent glioblastoma (GBM)
Intervention(s)	Sativex®, a nabiximols oromucosal spray
Key inclusion and exclusion criteria:	Ages eligible for study: ≥16 years<br>Sexes eligible for study: both<br>Accepts healthy volunteers: no
	Inclusion criteria: Karnofsky performance status ≥60, histological diagnosis of MGMT promoter methylated, IDH wild type GBM, a minimum of 3 cycles of adjuvant temozolomide received
	Exclusion criteria: Other active malignancy requiring treatment, pregnant or breastfeeding patients, personal history of psychotic illness
Study type	Interventional
	Allocation: randomised, double-blind
	Primary purpose: Efficacy
	Phase II

Data category	Information
Date of first enrolment	08-Mar-2023
Target sample size	234
Recruitment status	Open
Primary outcome(s)	Overall survival time defined as the time in whole days from date of randomisation to the date of death from any cause
Key secondary outcome(s)	Overall survival at 12 months defined as the time in whole days from date of randomisation to the date of death from any cause within the first 12 months<br>Progression-free survival time defined as the time in whole days from date of randomisation to the date of the first documented evidence of disease progression or death (from any cause)<br>Health related quality of life assessed at screening, and then every eight weeks until end of treatment, assessed using EORTC QLQ-C30, EORTC BN20, single items from the EORTC item library, and the EQ-5D-5L

3

Appendix 3: ARISTOCRAT trial patient summary and information sheet

To be printed on local hospital headed paper

ARISTOCRAT

A randomised controlled phase II trial of temozolomide with or without cannabinoids in patients with recurrent glioblastoma

You are being approached to consider participation in a clinical trial called ARISTOCRAT because you have previously been diagnosed with glioblastoma and, unfortunately, your recent MRI scan has shown that it has started to grow again. Your doctor has recommended further treatment for your disease and feels that you are suitable to consider taking part in this trial.

What is the purpose of this trial?

Glioblastoma multiforme is a type of brain tumour. When it is first diagnosed, patients are usually treated with surgery and then a combination of radiotherapy and chemotherapy with temozolomide (TMZ).

Unfortunately, although this often slows or stops the disease from growing for a period of time, in most cases, usually a few months after the end of the original treatment, the tumour starts to grow again. This can be detected by an MRI scan. When this happens patients may experience new symptoms or a repeat of previous symptoms.

There are few treatments available that work well at this stage to slow the growth of the tumour. Therefore, we need to develop new and better treatments to make patients live longer and feel better.

The ARISTOCRAT trial is investigating whether adding a second drug (Sativex, a cannabinoid or cannabis-based medicine) to the drug already used, TMZ, works better than TMZ alone. TMZ is the chemotherapy drug that you had before, both with radiotherapy and then afterwards by itself.

We are aiming to include 234 patients with recurrent glioblastoma to find out whether the addition of Sativex to standard TMZ treatment improves how long patients live, delays the growth of their tumour and/or improves their quality of life.

What happens next?

If you are interested in taking part and would like more detailed information about the ARISTOCRAT trial, then please ask your doctor.

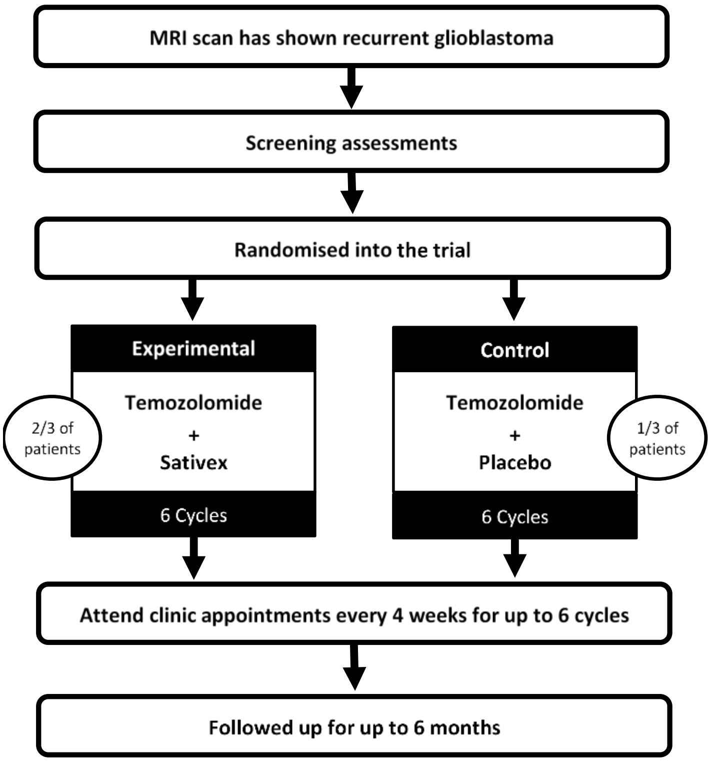

The diagram above shows an overview of the ARISTOCRAT trial.

A randomised controlled phase II trial of temozolomide with or without cannabinoids in patients with recurrent glioblastoma

Patient Information Sheet

To be used for patients over 16 years of age considering entering the ARISTOCRAT trial

We invite you to take part in a clinical trial

We would like to invite you to take part in an academic clinical trial, called ARISTOCRAT, run by the University of Birmingham, supported by The Brain Tumour Charity.

Before you decide whether you would like to take part in this trial, we would like you to understand:

- Why the trial is being done.
- What it would involve for you.

A member of the team at your local hospital involved in this trial will go through this information sheet with you and give you a copy to take away with you to discuss with friends and/or relatives, if you wish. They will answer any questions that you may have. If there is anything that is not clear within the information sheet, or you would like more information, please ask your medical team.

Take your time to decide whether or not you wish to take part. If you decide not to take part your doctors will continue to treat you in line with standard treatment and it will not affect the quality of your care.

Contents

Part 1: What is this clinical trial about?

1. Why are we doing this trial?

Part 2: What will happen to me if I take part?

1. What will I need to do if I take part?
2. What are the possible benefits and risks of taking part?
3. What happens at the end of the trial?
4. Will anybody get paid if I take part?
5. What if there is a problem?
6. Will my taking part in this trial be kept confidential?

Part 3: What else do I need to know about taking part in this clinical trial?

1. More information about taking part
2. Who should I contact for further information

Important things that you need to know

We want to improve the outcomes for people like yourself who have been diagnosed with a worsening of their brain tumour. In this trial, we want to find out if treatment works better if we add a second drug (Sativex) to the drug we already use as standard treatment (temozolomide, TMZ).

We will do this by:

- Looking at how well patients respond to the treatments.
- Assessing their health and quality of life during the trial, and afterwards.

This document is provided to help you understand what treatments will be given in the trial and provides answers to questions about the aims of the trial and what will happen to you if you take part.

Abbreviations

• DNA	Deoxyribonucleic Acid
• DVLA	Driving and Vehicle Licensing Agency
• GBM	Glioblastoma
• MRI	Magnetic Resonance Imaging
• MHRA	Medicines and Healthcare Products Regulatory Agency
• NHS	National Health Service
• REC	Research Ethics Committee
• TMZ	Temozolomide

Definitions

• Cannabinoid	Cannabis-based medicine
• Double blind	A trial where neither the researchers nor the patients know which treatment they are getting
• Glioblastoma	A type of brain tumour, sometimes referred to as GBM
• Placebo	A dummy treatment which will have no effect
• Randomisation	A random allocation of patients into one of several treatment groups.
• Standard of care	Typical treatment for a patient following a diagnosis of a brain tumour. Normally this is surgery followed by chemotherapy and radiotherapy.

We have tried to make this document readable and understandable by patients by involving patient representatives in writing this document. However, due to the nature of the trial, and the legal and regulatory requirements to include certain information, we are aware that some of the wording can seem complex. This makes it especially important that you ask your research team about anything that you do not fully understand.

If you have any further questions, you are very welcome to contact your medical team.

Part 1:
What is this clinical trial about?

1. Why are we doing this trial?

What is the purpose of this trial?

Glioblastoma multiforme is a type of brain tumour. When it is first diagnosed, patients are usually treated with surgery and then a combination of radiotherapy and chemotherapy with temozolomide (TMZ).

Unfortunately, although this often slows or stops the disease from growing for a period of time, in most cases, usually a few months after the end of the original treatment, the tumour starts to grow again. This can be detected by an MRI scan. When this happens patients may experience new symptoms or a repeat of previous symptoms.

There are few treatments available that work well at this stage to slow the growth of the tumour. Therefore, we need to develop new and better treatments to make patients live longer and feel better.

This trial is investigating whether adding a second drug (Sativex, a cannabinoid or cannabis-based medicine) to the drug already used, TMZ, works better than TMZ alone. TMZ is the chemotherapy drug that you had before, both with radiotherapy and then afterwards by itself.

In a small trial already completed investigating the combination of TMZ and Sativex, there were some interesting results suggesting that taking both drugs together were safe to give and may have an effect on the growth of brain tumours. To see if this treatment does work we need to do a much larger trial and compare the new treatment (TMZ plus Sativex) to TMZ (TMZ plus placebo).

We are aiming to include 234 patients with recurrent glioblastoma to find out whether the addition of Sativex to standard temozolomide treatment improves how long patients live, delays the growth of their tumour and/or improves their quality of life.

Why have I been invited to take part?

You have been invited to take part in this trial because you have previously been diagnosed with glioblastoma and, unfortunately, your recent MRI scan has shown that it has started to grow again. Your doctor has recommended further treatment for your disease and feels that you are suitable to consider taking part in this trial.

Do I have to take part?

No, you do not have to take part. It is up to you to decide whether to join the trial. A member of the local research team will describe the trial and go through this information sheet with you. If you decide to take part, you will be asked to sign a consent form to show you have agreed to participate. You are free to withdraw at any time, without giving a reason. A decision not to take part or to withdraw later will not affect the standard of care you receive going forwards.

If you decide not to take part, no data will be collected about you. If you decide to withdraw from the trial later, please read Part 3 to understand what will happen to the information collected about you during the trial.

What are the treatments being tested?

Temozolomide:

Temozolomide is a type of chemotherapy, which works by binding to the cancer cell DNA and stopping cancer cells from dividing and growing. It is the standard drug used to treat glioblastomas.

Sativex:

Sativex is a mouth spray that contains cannabis extracts or cannabinoids. It has been licensed in the UK as a treatment for the neurological condition multiple sclerosis. It is manufactured (made) and supplied by the pharmaceutical company GW Pharma. Sativex is sometimes referred to by the name Nabiximols.

Randomisation and Blinding:

This trial is called a 'double-blind randomised trial'. This means that for those who enter the trial, a computer will randomly put each participant into one of two groups, called 'arms'. Participants in one 'arm' will be given the drug combination of temozolomide and Sativex. Participants in the other 'arm' will be given temozolomide and a placebo – a 'dummy' drug that is also a mouth spray and looks like Sativex but does not contain any active drug. Participants will be randomised to the two arms on a 2:1 ratio, which means that there is a greater chance of receiving temozolomide with Sativex than temozolomide with placebo.

If you take part you will not know which 'arm' you are in. Your doctor and medical team as well as the trial team will also not know this (this is the double blind part). It will only be at the end of the trial that the researchers will uncover this information to see if there are different outcomes between the two groups and to see if the treatment in one arm works better than the treatment in the other arm overall.

Participants sometimes ask, for this type of clinical trial, why all the patients cannot be given the trial drug (Sativex in this trial). This is because of the very small number of patients in the original trial which means that we cannot be sure that any potential benefit seen in patients who received Sativex was not by chance. This can only by doing a much larger randomised trial comparing the effects of temozolomide with Sativex against temozolomide with a placebo. It is important to remember that you will still receive temozolomide in the same way that you would if you were not on the trial.

This completes Part 1 of the Information Sheet

If the information in Part 1 has interested you and you are considering participation, please read the additional information in Parts 2 and 3 before making any decision.

Part 2:
What will happen to me if I take part?

1. What will I need to do if I take part?

If you decide to take part in this trial, you will be given this information sheet to keep and you (and your trial doctor) will be asked to sign a consent form to show that you have agreed to take part in this trial. You will be given a copy of the signed consent form to take home with you.

We will then need to check that it is appropriate for you to take part in the trial. This is called screening. It is possible that even after deciding that you want to take part that you may not be able to. This is because all trials have criteria that must be met for patients to be able to take part; unfortunately, these aren't things you can change. Decisions about your treatment are independent of your participation in the ARISTOCRAT trial and will continue to be made in discussion with you and your doctor so that you receive the best care available.

The diagram below shows a summary of the ARISTOCRAT trial:

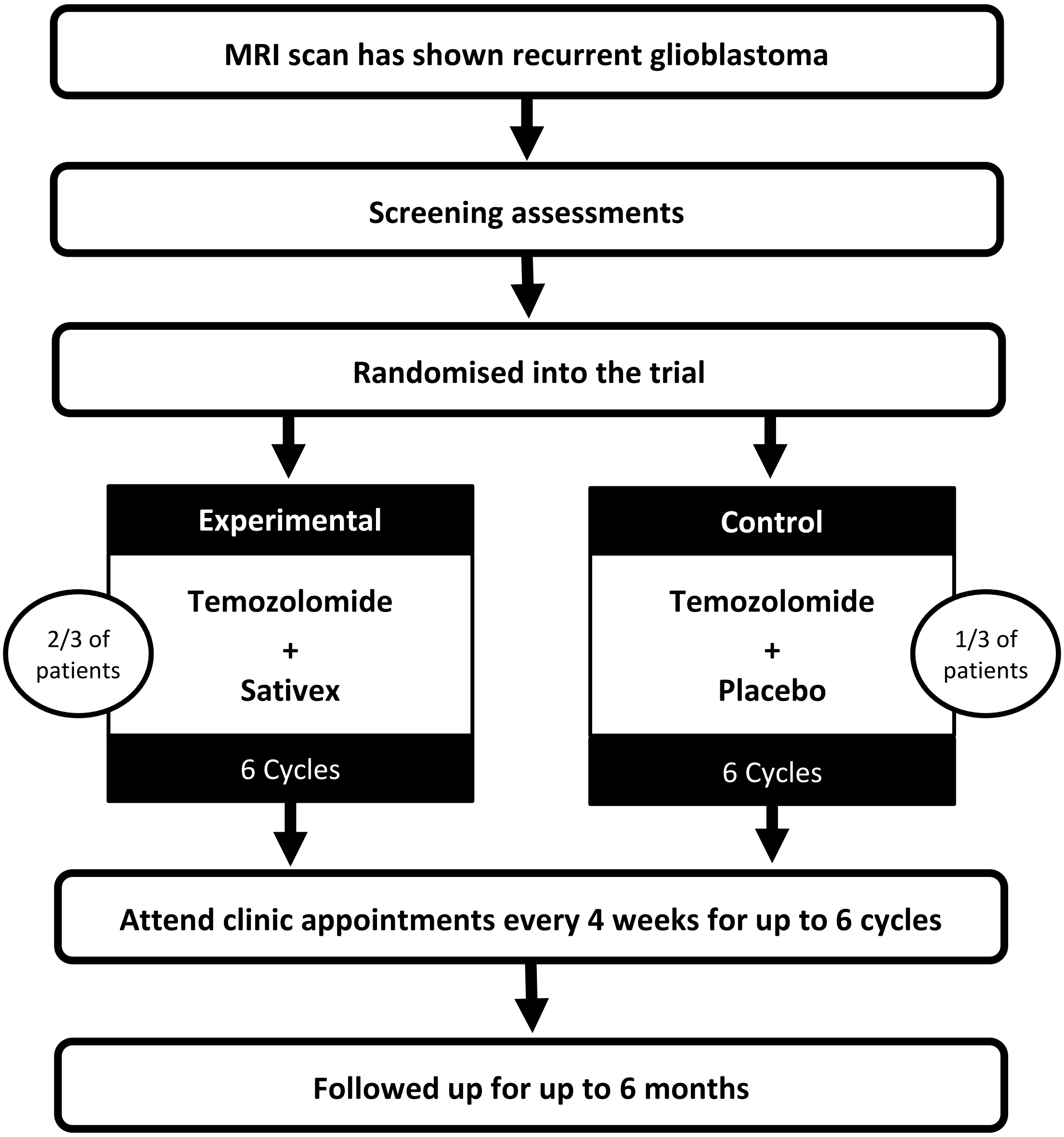

Summary of Assessments

	Before Treatment		Treatment period								End of Treatment	Follow Up		
**Assessment / Activity**	**Screening**	**Randomisation**	**Week 0** (Cycle 1)	**Week 4** (Cycle 2)	**Week 8** (Cycle 3)	**Week 10**	**Week 12** (Cycle 4)	**Week 16** (Cycle 5)	**Week 20** (Cycle 6)	**Week 22**	**Week 24**	**Week 28**	**Week 30**	**Standard of Care**
**Clinical Assessments**														
Electrocardiogram	✓													
Medical history	✓													
Medications monitoring	✓		✓	✓	✓		✓	✓	✓		✓	✓		✓
Performance status	✓		✓	✓	✓		✓	✓	✓		✓	✓		✓
Physical check-up	✓		✓	✓	✓		✓	✓	✓		✓			
Side effects monitoring			✓	✓	✓		✓	✓	✓		✓	✓		✓
Vital signs	✓		✓	✓	✓		✓	✓	✓		✓			
**Laboratory Assessments**														
Blood samples	✓		✓	✓	✓		✓	✓	✓		✓			
Pregnancy test (Female patients only)	✓		✓	✓	✓		✓	✓	✓		✓			
Urine test for cannabinoid use			✓		✓						✓			
**Patient Questionnaires**														
Patient diary			✓	✓	✓		✓	✓	✓					
Quality of Life Questionnaires			✓		✓			✓			✓			
**Treatment**														
Temozolomide & Sativex/placebo			✓	✓	✓		✓	✓	✓					
**Tumour Assessment**														
MRI scan	✓					✓				✓			✓	✓

Before you begin the trial:

Screening:

You will need to have the following screening assessments to ensure that it is appropriate and safe for you to take part.

- A physical check-up, including measuring your height and weight and a neurological assessment.
- Performance status
  This is a measure of your general health and how your disease affects your daily routine.
- Vital signs including your blood pressure and pulse.
- Electrocardiogram (ECG)
  This is an electrical recording of your heartbeat, taken to check the health of the heart. It involves small pads being attached to your chest, with wires attaching them to a machine that produces a printout of the beat. This will not hurt or harm you and should only take about 5 minutes.
- Pregnancy test (if female and of child-bearing potential).
- Routine blood tests
- MRI scan of the brain
  This is a type of scan that creates detailed pictures of the brain using magnetism and radio waves. MRI scans produce pictures from angles all around the body and show up soft tissues very clearly. This will tell us where your tumour is located and what size it is.
  You will probably have had this type of scan before. Before having the scan, you may be given a special dye called a contrast medium to help improve the quality of the images. During the scan, you will usually lie on your back on a couch that can slide into the MRI scanner.
  The scanner consists of a ring that rotates around a small section of your body and takes pictures as you move through it. The scanner makes a very loud clanging sound throughout the scan. You wear headphones to protect your hearing. You can also listen to music. Keeping your eyes closed can help. The scan will take between 15-90 minutes.
- Your trial doctor will ask your questions about your medical history and details of any other medication you are currently taking.

The physical check-up, performance status, vital signs, routine blood tests and an MRI scan would be provided anyway as part of your regular care. The ECG and pregnancy test are extra tests as part of this trial. If you have had some of them recently, they may not need to be repeated.

The screening period may last up to 3 weeks while results are collected. Results of these tests and scans will be used to confirm if you are suitable and it is safe for you to enter the trial (i.e. if you are eligible). If you are eligible, your trial doctor will randomise you to take part in the trial with the ARISTOCRAT Trial Office (at the Cancer Research UK Clinical Trials Unit (CRCTU), University of Birmingham) and you will be assigned a unique Trial Number.

Tessa Jowell BRAIN MATRIX

Some ARISTOCRAT patients may already be enrolled on the Tessa Jowell BRAIN MATRIX study, a separate study for brain tumour patients, which is also run by the University of Birmingham. This will streamline your entry into ARISTOCRAT and will provide your doctor with a detailed diagnosis of your disease.

BRIAN

Patients joining ARISTOCRAT will also be able to support a study, called BRIAN (the Brain tumouR Information and Analysis Network), run by The Brain Tumour Charity.

BRIAN allows people to record their experiences of having a brain tumour using a mobile phone or web app. This is to help doctors and researchers get a better understanding of how having a brain tumour affects the quality of life of patients who are living with a brain tumour or caring for someone who has a brain tumour. Your doctor will talk to you about participating in this additional study, which does not involve any change in your planned treatment. In the future, it is hoped that by analysing information collected by BRIAN together with all the other data collected from patients participating in ARISTOCRAT, it will be possible to provide better overall care and support to patients and their families.

You can find out more about BRIAN and how to take part at the Brain Tumour Charity's website: https://www.thebraintumourcharity.org/living-with-a-brain-tumour/brian/

You are welcome to take part in BRIAN even if you do not wish to take part in this trial. Similarly, you can still participate in ARISTOCRAT if you do not want to join BRIAN.

During the trial:

Trial Treatment

Once you have been randomised into the trial, your treatment will commence in the next few days. Treatment may continue up to a maximum of 6 cycles, as long as you are not suffering unacceptable side effects and it appears to be working. The side effects experienced with these treatments are listed later.

Temozolomide

During the trial, temozolomide is taken by mouth once daily for 5 days at the start of each 28 days (4 weeks) cycle, up to a maximum of 6 cycles. You will take a dose 150 mg/m^2 for cycle 1, increasing to 200 mg/m^2 for Cycles 2-6. This is the standard dose you would receive outside of ARISTOCRAT trial.

Temozolomide is given as capsules to be taken at home. It should be stored below 30°C. You should take it at the same time each day. You should swallow the capsule whole with a glass of water and on an empty stomach.

If you vomit after taking the dose, a second dose should not be taken that day.

If at the end of Day 5 you have any capsules left over because you missed a dose, these should not be taken. Instead, return these unused capsules to your trial doctor or research nurse at your next clinic visit.

Sativex/placebo

During the trial, Sativex/placebo is a spray that is taken by spraying into the mouth every day for up to 6 cycles.

Unopened Sativex/placebo vials should be stored upright in a refrigerator at 2°C to 8°C. If it is not stored in a refrigerator, it is unlikely to work. Once opened, store the Sativex/placebo vials in an upright position below 25°C. If the storage temperature once opened exceeds 25°C, then the vial can be stored in a refrigerator. You must not use Sativex after it has been open for 42 days.

You will spray Sativex/placebo in your mouth by pointing the nozzle under your tongue or at the inside of your cheek. Each time you use the spray, you should try to use a different area of your mouth. This is to avoid any discomfort and irritation developing in one place.

The amount of sprays you need each day depends on you as an individual. This will start as a single spray in the evening of day 1 of cycle 1. You will then gradually increase the number of sprays of Sativex/placebo by following the table below. You will do this until you reach the highest number of sprays with the fewest number of side effects or until you are using 12 sprayed doses each day. This may take a few days, or it may take up to 2 weeks. Once you have found the daily dose that works for you, carry on using this number of sprays each day. You can then spread your sprays evenly throughout the day. You must not use more than 12 sprays in one day.

A minimum of 3 sprays per day is recommended, but if you aren't able to do this you can still stay on trial, but your information might not contribute to the trial results.

Table 1: Sativex/placebo self-titration

Day	Number of sprays		
	**Morning** (between waking-up and 12pm)	**Afternoon** (between 4pm and bedtime)	**Total number of sprays each day**
**1**	0	1	1
**2**	0	1	1
**3**	0	2	2
**4**	0	2	2
**5**	1	2	3
**6**	1	3	4
**7**	1	4	5
**8**	2	4	6
**9**	2	5	7
**10**	3	5	8
**11**	3	6	9
**12**	4	6	10
**13**	4	7	11
**14**	5	7	12

How to take Sativex/placebo:

You start off taking one spray a day in the afternoon/evening, which should be taken at any time between 4pm and bedtime. When the morning spray is introduced, it should be taken at any time between waking up and 12pm. You must not use more than one spray at the same time and you should always leave at least 15 minutes between sprays.

If you forget a spray, use a spray as soon as you remember as long as you do not exceed the maximum number of 12 sprays in one day. You must not use 2 sprays at the same time to make up for a missed spray.

You should not overexert yourself during the first couple of sprays until you know how it affects you. You should not breathe in the spray and avoid getting the spray into your eyes. If it get in your eyes by accident, you should rinse them as soon as possible with water. You should avoid taking it around

children, animals, naked flames or heat sources. Sativex/placebo should be stored out of reach of children.

You can use Sativex/placebo with and without food. However, you should try, as far as possible, to take the drug the same time in relation to food so that you get the same effect each time. Drinking alcohol should be avoided whilst using Sativex, especially at the beginning of treatment, as using them together they may increase the risk of falls and other accidents.

A carer may help with the spray if you are unable to do so yourself.

Foreign travel with Sativex:

If you plan to go abroad, please check that it is legal for you to take this medication with you. This is because Sativex is a controlled drug and therefore its legal status will vary between countries. Your doctor should be able to provide you with a letter explaining your participation in the trial and that this is a prescribed medication if you require this.

What does Sativex contain?

Sativex contains cannabis extracts or cannabinoids

Sativex also contains up to 40 mg of alcohol (ethanol) per dose. The amount of alcohol in the maximum daily dose (12 sprays per day) is about the same as found in two teaspoons (10 ml) of beer and about one teaspoon (5 ml) of wine. The small amount of alcohol in this medicine will not have any noticeable effects.

A decision to not take part because Sativex contains cannabis extracts and alcohol will not affect the standard of care you receive going forwards.

Sativex also contains 52 mg of propylene glycol, which may cause irritation.

Clinic Appointments

You will be reviewed by your trial doctor and their team while on the trial. You will need to attend clinic appointments every 4 weeks whilst receiving treatment so we can assess your progress. These visits should fall within what would be your routine care.

The research team will perform the following assessments at each visit to see how the trial medication is affecting your body.

- A physical check-up, including measuring your weight and a neurological assessment.
- Performance status.
- Vital signs including your blood pressure and pulse.
- Pregnancy test (if female and of child-bearing potential).
- Routine blood tests to assess ongoing safety.
- Record any medication you are currently taking and any side effects that you have experienced since your last visit.
- A urine test for cannabis/cannabinoid use before starting trial treatment at Cycle 1 and at Cycle 3. This is to check that cannabis is not being taken outside of the trial.

All of these tests and assessments, except for the urine test, would be carried out routinely in standard of care.

During trial treatment, you will also attend for an MRI scan of the brain on weeks 10 and 22, which will be used to assess the growth of your tumour. This is the same as for standard care.

You should tell your doctor of any new health problems which occur while you are taking part in this trial. Your doctor will be kept updated by the trial team about any serious, unexpected reactions that occur during the trial and may discuss these with you.

Other requirements

Patient Diary

Whilst you are taking the trial drugs you will be given a Patient Diary in which to record when you take your medication. Your nurse will show you how to complete it. This will help you to remember if you have taken your doses and to record any side effects. A carer may assist you with completing the diary.

If you accidentally miss a dose, it is important that this is recorded so that your doctor can see what medication you have taken. You will need to bring this diary to your clinic visits. You will be issued with a new diary for each cycle. In addition, you must return any used or empty Sativex/placebo sprays and any unused capsules to your trial doctor or research nurse when you attend clinic.

Health-Related Quality of Life Questionnaires

We know that brain tumours can impact many areas of your life and we want to understand this better. To do this you will be asked to complete a 'Quality of Life' booklet that consists of two short questionnaires designed to collect information about symptoms you are experiencing and how you are feeling. This information is important to allow us to assess how the trial treatment is impacting your day-to-day activities.

Your trial doctor or research nurse will explain to you how to complete these questionnaires. You will be asked to complete the questionnaires before your first dose of trial treatment, before cycles 3 and 5 while on treatment and when you finish treatment. The questionnaires should take no longer than 20 minutes to complete. A carer may assist you with completing the questionnaire, but the answers should be your own.

Patient Identification Card

You will be issued with a Patient Identification Card on which your patient Trial Number and the trial treatment will be recorded. This will not disclose if you are receiving Sativex or placebo. Emergency contact numbers that you can use if you feel unwell at any time are also recorded on it. You must carry the Patient Identification Card with you at all times and present it to a doctor if you are admitted to a hospital. This is especially important for this trial due to the blinded treatment allocation (see Part 1 – Randomisation and Blinding).

After you stop taking the trial drugs:

You will stop taking the trial drugs when you have completed 6 cycles of treatment. Your trial doctor may advise you to stop treatment early if you are experiencing unacceptable side effects or if it does not appear to be working. You will also stop treatment if you withdraw from the trial.

When you have finished taking the trial drug you will need to come to the clinic for an end of treatment visit. During this visit, your research team will perform the following assessments:

- A physical check-up, including measuring your weight and a neurological assessment.
- Performance status.
- Vital signs including your blood pressure and pulse.
- Pregnancy test (if female and of child-bearing potential).
- Routine blood tests to assess ongoing safety
- Record any medication you are currently taking and any side effects that you have experienced since your last visit.
- A urine test for cannabis/cannabinoid use.

All of these tests and assessments, except for the urine test, are the same as for standard care.

You will continue to be followed up for up to 6 months to see how the trial affected your health. You will need to attend clinic every 4 weeks during this period. This is the same as standard care. During

these visits your performance status will be assessed and you will be asked about any side effects you may have experienced and any additional medication you are currently taking.

You will also attend for an MRI scan of the brain at week 30 and then every 3 months from the end of treatment until the end of follow up, which will be used to assess the growth of your cancer. This is the same as for standard care.

What will I have to do?

If you decide to take part in this trial, you will be required to:

- ✓ Sign a consent form to enter the trial.
- ✓ Take the trial medication as directed.
- ✓ Return any unused capsules and any used or empty Sativex/placebo sprays when you attend clinic.
- ✓ Keep all scheduled medical and imaging appointments.
  (There are no additional appointments or scans as part of this trial).
- ✓ At routine clinic visits your vital signs, weight and performance status (measure of your general health and how your disease affects your daily routine) will be taken.
- ✓ Provide routine blood samples to assess ongoing safety.
- ✓ Tell your trial doctor about any side effect, injury, symptom or complaint you experience, including any unplanned hospital admissions, when you attend clinic appointments.
- ✓ Tell your trial doctor about any other medication that you are taking, even if it is medicine you buy without a prescription or is a natural or herbal remedy. This is because some medicines may increase the risk of side effects with temozolomide and Sativex and any prohibited medication must not be taken throughout the trial. Your trial doctor will tell you about any prohibited medications.
- ✓ Complete a diary to record when you took your trial medication and bring it with you to your clinic appointments.
- ✓ Complete Health-Related Quality of Life questionnaires when you join the trial and when requested during and after trial treatment.
- ✓ Use adequate contraception during the trial and for at least 6 months after trial treatment has finished.

What are the alternatives for treatment?

If you choose not to take part in this trial, your doctor will discuss with you if any alternative treatment options are suitable for you.

How long will I be in the trial?

You will receive the trial drugs for up to 6 cycles and will be followed up for up to 6 months. We would like to continue to collect data on you for up to 18 months where possible.

2. What are the possible benefits and risks of taking part?

What are the possible benefits of taking part?

We cannot promise that you will benefit directly from participating in this trial. It is possible that the treatment you receive will be more effective or have different side effects. However, we will not know this until the results of the trial are available.

All the information that we get from this trial will help improve the treatment of patients with recurrent glioblastoma in the future.

What are the possible disadvantages and risks of taking part?

We want to try and improve the outcome for patients with recurrent glioblastoma and believe that the trial treatments being used in this trial may do this. However, it is possible that the treatments being used in this trial may not show any benefit over the current UK standard practice, or that there may be more side effects.

Current government guidelines and local safety procedures in relation to the ongoing COVID-19 pandemic will be adhered to in order to minimise any risk of exposure to the virus.

In addition to side effects you may experience as a result of the trial treatment as discussed below, there are other potential risks which are associated with the trial:

Blood samples

Routine blood samples will be taken during the trial as outlined above. You may experience a little discomfort when the needle is put into your vein to take the blood sample. There may be a slight pain, a small amount of bleeding, discolouration, or bruising at the site where the needle is inserted (but this will clear after a week or two). There is also a risk of infection or phlebitis (abnormal blood clot), but these rarely happen (less than 1 in 100 patients). Blood samples will be grouped as much as possible to minimise the number of times blood is drawn.

Effects on operating machinery

Temozolomide and Sativex may affect your ability to use machines safely. This is because the drugs may cause you to feel tired or dizzy, which may impair your judgement and performance of skilled tasks. You should not operate machinery or tools if you have side effects such as tiredness, sleepiness or dizziness.

Patients with recurrent GBM are not permitted to drive and more information can be found on the DVLA website regarding this https://www.gov.uk/health-conditions-and-driving.

Mental health

The health-related quality of life questionnaires will also ask questions about your mental health due to the potential side effects of Sativex. You may find some of these questions upsetting and difficult to answer. If any of the questions upset you, then please speak to your doctor, nurse or another healthcare professional.

Harm to unborn child and pregnancy

The effects of temozolomide and Sativex may be harmful to an unborn child and should not be given to women who are pregnant or breastfeeding. It is therefore essential that both men and women taking the trial drugs must agree to use a reliable form of contraception during the trial.

Information for women

You should not take part in this trial if you are pregnant, breastfeeding or may become pregnant during the trial period. If you are female and of childbearing potential, you will have a pregnancy test during screening.

You must agree to use a reliable form of contraception during the trial and for at least 6 months after the trial treatment has finished. Acceptable forms of contraception are barrier methods (e.g. condoms) for males and hormonal methods (e.g. contraceptive pill, implant or Minera coil) for females.

Information for men

Male patients with partners who are pregnant or who could become pregnant (i.e. women of child-bearing potential) must agree to use a reliable form of contraception during the trial, and for at least 6 months after the trial treatment has finished. Acceptable forms of contraception are barrier methods (e.g. condoms) for males and hormonal methods (e.g. contraceptive pill, implant or Minera coil) for females.

Your doctor will talk to you about potential sperm donation before you start treatment and you should not be involved in sperm donation during treatment.

Monitoring Pregnancies

If you or your partner do become pregnant whilst you are on the trial treatment or up to 6 months after your last dose , please tell your trial doctor immediately. It will be important to monitor the outcome of the pregnancy for any congenital anomalies or birth defects. If this occurs, then you or your partner will be given a Release of Medical Information Form. If you or your partner are happy to provide information regarding the outcome of the pregnancy, you should sign this form and return it to your trial doctor or research nurse. Once you have returned the Release of Medical Information Form, your trial doctor or research nurse will provide details of your pregnancy back to the ARISTOCRAT Trial Office. This will be followed up for up to 10 years.

What are the side effects of the treatment?

You may have side effects while on the trial. All treatments can have side effects (known as adverse events or toxicities). Everyone taking part in the trial will be carefully monitored for any side effects. However, doctors don't know all the side effects that may happen. Side effects may be mild or very serious. Your health care team may give you medicines to help reduce side effects or in some cases treatment may be delayed.

In the event of severe side effects and at the advice of your trial doctor, trial treatment will be stopped until side effects have eased. Treatment may also be interrupted temporarily. If the side effects have not sufficiently reduced, trial treatment will be permanently stopped.

A list of currently known side effects with a direct relationship to temozolomide and Sativex in patients with recurrent glioblastoma, found from previous studies, are listed below:

Temozolomide

Very common side effects seen in patients treated with temozolomide (more than 1 in 10 patients):

- Loss of appetite
- Seizures (fits)
- Weakness on one side of the body
- Difficulty in speaking
- Headache
- Constipation or diarrhoea
- Feeling or being sick
- Rash
- Hair loss
- Tiredness

Common side effects seen in patients treated with temozolomide (more than 1 in 100 but less than 1 in 10 patients):

- Infections
- Sore throat
- Oral thrush
- Low white blood cell count, fever and infections
- Reduction in red blood cells and platelets
- Allergic reaction
- A build-up of fluid in your arms and legs
- High blood sugar levels
- Confusion, anxiety and memory loss
- Low mood
- Difficulty sleeping
- Difficulty speaking and concentrating
- Reduced consciousness
- Dizziness
- Numbness or tingling usually in your fingers and toes
- Sleepiness
- Changed sense of taste
- Shaking/tremor
- Blurred vision and eye pain
- Hearing changes such as ringing in your ears and hearing loss
- Problems with your balance
- Bleeding problems
- Blood clots
- High blood pressure
- Lung infection
- Difficulty breathing and coughing
- Sore mouth
- Indigestion
- Difficulty swallowing
- Skin rash, dry skin and itching
- Muscle weakness and stiffness
- Pain in different parts of your body such as your back, muscles, joints and tummy (abdomen)
- Difficulty controlling your bladder
- Fever and flu-like symptoms
- Liver changes
- Weight changes

There are other less common side effects of temozolomide. If you would like a list of these, please ask your doctor.

Sativex

Very common side effects seen in patients treated with Sativex (more than 1 in 10 patients):

- Dizziness
- Tiredness

Dizziness most frequently occurs in the first few weeks of treatment and if it occurs will be assessed by your trial doctor when you attend clinic at Cycle 2.

Common side effects seen in patients treated with Sativex (more than 1 in 100 but less than 1 in 10 patients):

- Change in appetite
- Low mood
- Feeling confused about time or place
- Exaggerated feeling of happiness
- Problems with your memory or having trouble concentrating
- Difficulty speaking
- Changed sense of change or dry mouth
- Lack of energy or feeling weak or generally unwell
- Sleepiness
- Blurred vision
- Constipation or diarrhoea
- Feeling or being sick
- Mouth problems including discomfort, pain or mouth ulcers
- Irritation where Sativex is sprayed
- Feeling abnormal or drunk
- Discomfort
- Loss of balance or falling over

Uncommon side effects seen in patients treated with Sativex (more than 1 in 1,000 but less than 1 in 100 patients):

- Tummy aches or cramps
- Feeling things that are not true (Delusions)
- Seeing things which aren't there (Hallucinations)
- High blood pressure (Hypertension)
- Noticeable heartbeat (Palpitations)
- Sore throat (Pharyngitis)
- Feeling like you are being threatened in some way, even if there is little evidence of this (Paranoia)
- Suicidal feelings
- Fainting
- Racing heartbeat (Tachycardia)
- Change in colour of teeth

There are other less common side effects of Sativex. If you would like a list of these, please ask your doctor.

3. What happens at the end of the trial?

At the end of the trial, or if you withdraw from the trial, your trial doctor will assess your symptoms and discuss your options and prescribe appropriate treatment.

If the academic body sponsoring the research trial decides to stop the trial before it has finished, your trial doctor will explain the reasons why and arrange appropriate care for you.

4. Will anybody get paid if I take part?

You will not receive any money or trial expense reimbursement for taking part in this clinical trial.

5. What if there is a problem?

If you have any concerns about your care during this trial or any possible harm you may suffer, you should inform your trial doctor immediately. More detailed information is given in Part 3 of this information sheet.

6. Will my taking part in this trial be kept confidential?

Yes. We will follow ethical and legal practice and all information about your participation in this trial will be kept confidential. The details of this are in Part 3 of this information sheet.

This completes Part 2 of the Information Sheet

If the information in Part 1 and Part 2 has interested you and you are considering participation, please read the additional information in Part 3 before making any decision.

Part 3:
What else do I need to know about taking part in this clinical trial?

1. More information about taking part

What if relevant new information becomes available?

Sometimes new information about the conditions and drugs being studied becomes available during the course of a research trial. If this happens, your trial doctor will tell you about it and discuss this with you. If you decide not to carry on, your trial doctor will make arrangements for your care to continue as normal. If you decide to continue with the trial then you may be asked to sign an updated consent form. If the trial is stopped for any other reason, your doctor will tell you and arrange your continuing care.

What if I decide that I don't want to carry on with the trial?

You are free to withdraw from this trial at any time. You do not have to give a reason for your decision and your future treatment will not be affected. Your trial doctor will discuss your treatment options with you and will offer you the most suitable treatment available.

However, if you were to withdraw from treatment, we would like your permission for your hospital to continue to send information on your health status and any further treatment you may have to the ARISTOCRAT Trial Office. You may withdraw from this data collection, although any data already collected up until your withdrawal will still be used for trial purposes.

Loss of mental capacity

Capacity means the ability to use and understand information to make a decision, and communicate any decision made. A person lacks mental capacity if their mind is impaired or disturbed in some way, which means they're unable to make a decision at that time. This is known as loss of mental capacity.

If you lose mental capacity whilst participating in the trial, then your carer will be responsible for deciding if you would like to continue on the trial or if you would have wanted to be withdrawn from the trial.

If they decide to withdraw you from the trial, then they do not have to give a reason for your decision and your future treatment will not be affected. There will be no further trial procedures performed or data collected, although any data already collected up until your withdrawal will still be used for trial purposes. If they decide that you would like to continue on the trial, then they will be asked to provide trial related information, such as any side effects experienced, on your behalf.

What if there is a problem?

In the event that something does go wrong and you are harmed because of taking part in the trial, and this is due to someone's negligence, then you may have grounds for claiming compensation from the Sponsor of the trial (the University of Birmingham) or the NHS Trust who treated you but you may have to pay your legal costs. The Sponsor does not hold insurance against claims for no-fault injury caused by participation in this trial and they cannot offer any indemnity.

NHS Trust and non-Trust Hospitals have a duty of care to patients treated, whether or not the patient is taking part in a clinical trial, and the normal NHS complaints mechanism will still be available to you (if appropriate).

What will happen to any samples I give?

Routine safety blood samples will be analysed at your hospital laboratory to see how the trial medication is affecting your body. Urine for pregnancy testing will be analysed by your hospital. Any routine blood samples and urine collected for pregnancy testing will be destroyed after analysis. Routine blood and urine samples will be labelled in accordance to standard local practices at your hospital.

Urine samples to test for cannabinoid use will be sent to Matrix Diagnostics for analysis. Any urine samples remaining at the end of this analysis will be destroyed. Urine samples for cannabinoid testing will be labelled with only your trial number.

We may also request tissue samples from your tumour that are already stored in your hospital pathology department; these would be analysed as part of research associated with this trial or be biobanked for future ethically approved studies.

If funding becomes available, we may wish to collect additional samples for translational studies to better understand your cancer and the effect the treatment may be having on it. We may also wish to store these samples for further research.

When you are consented to take part in the trial, you will be asked to provide optional consent for you to be approached about collecting further samples. If you agree to this and funding becomes available, you would be provided with further information by your trial doctor and asked to complete a separate consent form. Again, this would be optional and there is no requirement for you to agree with this to participate in the trial.

Will any genetic tests be done?

No genetic analysis of your samples will be performed in this trial.

Will my taking part in this trial be kept confidential?

Yes. All information collected about you for this trial will be subject to the General Data Protection Regulation (GDPR) and the Data Protection Act 2018 and will be kept strictly confidential. University of Birmingham is the Sponsor for this trial based in the UK. We will be using information from your medical records in order to undertake this trial and will act as the data controller for this trial. This means that we are responsible for looking after your information and using it properly. University of Birmingham and the NHS will keep identifiable information about you for at least 25 years after the trial has finished, allowing the results of the trial to be verified if needed.

All information collected by the Sponsor will be securely stored at the Cancer Research UK Clinical Trials Unit (CRCTU) at the University of Birmingham (ARISTOCRAT Trial Office) on paper and electronically and will only be accessible by authorised personnel. The only people at the University of Birmingham who will have access to information that identifies you will be people who manage the trial or audit the data collection process. With your permission, your trial doctor will notify your GP that you intend to participate. They will also send a copy of your signed Informed Consent Form in the post to the ARISTOCRAT Trial Office to ensure that the correct consenting procedure has been carried out. This will have your name and signature on it.

The NHS will use your name and contact details to contact you about the research trial, and make sure that relevant information about the trial is recorded for your care, and to oversee the quality of the trial. Your trial doctor or research nurse may also need to send a copy of your Informed Consent Form to other healthcare professionals (e.g. your GP or NHS pathologist) to prove that you have given consent to take part in the trial before they will provide information or tumour samples for example.

All information will be treated as strictly confidential and nothing that might identify you will be revealed to any third party other than those involved in your treatment or organisation of samples (e.g. staff at University of Birmingham and University of Leeds). It may be necessary to send

information about you, such as trial number and date of birth, to the collaborating company GW Pharma Ltd, Matrix Diagnostics who are processing the urine cannabinoid testing and to the BRIAN databank (IRAS ID: 237931) run by The Brain Tumour Charity. This is for your, and others' protection to track the safety of the treatments used. They have the same duty of confidentiality to you as all other research trial personnel. Data may be transferred outside of the UK, as we will be providing safety data and the end of trial report to GW Pharma which, despite being UK based, is a subsidiary of Jazz Pharmaceuticals based in the USA & Ireland. Samples will not be transferred outside the UK. By taking part in the trial you will be agreeing to allow research staff at your hospital, and from the ARISTOCRAT Trial Office to look at the trial records, and this includes your medical records. It may be necessary to allow authorised personnel from the University of Birmingham and/or NHS bodies to have access to your medical and research records. This is to ensure that the trial is being conducted to the highest possible standards. We may also collect different aspects of your health data from the NHS and other Department of Health organisations, in addition to your medical records, for the purposes of long term follow up.

All individuals who have access to your information have a duty of confidentiality to you.

If you choose to withdraw from the trial treatment, we would still like to collect relevant information about your health, as this will be invaluable to our research. If you have any objection to this, please let your trial doctor know.

How we will use information about you?

We will need to use information from your medical records for this research project. This information will include you:

- Your name
- Initials
- Date of birth
- Ethnicity
- NHS/CHI number and hospital number
- Histopathology number to collect your tissue samples

People who do not need to know who you are will not be able to see your name or contact details. Your data will have a code number instead. We will keep all information about you safe and secure.

Once we finish the study, we will keep some of the data so we can check the results. We will write our reports in a way that no-one can work out that you took part in the study.

What are your choices about how your information is used?

You can stop being a part of the study at any time, without giving reason, but we will keep information about you that we already have. We need to manage your records in specific ways for the research to be reliable. This means that we won't be able to let you see or change the data we hold about you.

Where can you find out more about how your information is used?

You can find out more about how your information is used by:

- Accessing the CRCTU Privacy Policy available on our website: www.birmingham.ac.uk/research/crctu/data-protection.aspx.
- Asking a member of the research team
- Contacting the University's Data Protection Officer by email: dataprotection@contacts.bham.ac.uk
- Or by calling us on 0121 414 6788

What will happen to the results of the trial?

Your trial doctor will receive regular safety updates throughout the trial. The final results from the trial will be published in medical journals but no individual patients will be identified. The results will help to decide how to treat recurrent brain tumours in the future. When you are consented to take part in the trial, you will be asked to provide optional consent to receive a lay summary of the results at the end of the trial. You will be able to get a copy of the published results by asking your doctor at the end of the trial.

Who is organising and funding the research?

This research trial is being carried out by a network of doctors across the UK. The trial is sponsored and insured by the University of Birmingham (UoB) and coordinated by the Cancer Research UK Clinical Trials Unit at the University of Birmingham. The trial is being funded by The Brain Tumour Charity and the Sativex/placebo drug supplied by the pharmaceutical company GW Pharma Ltd. Your trial doctor will not be paid for including you in this trial.

UoB has in place Clinical Trials indemnity coverage for this trial with a Limit of Indemnity of £10,000,000 which provides cover to UoB for harm which comes about through the University's, or its staff's, negligence in relation to the design or management of the trial and may alternatively, and at UoB's discretion provide cover for non-negligent harm to participants.

The NHS Trust has a duty of care to its patients, in the event of clinical negligence being proven, compensation will be available via the NHS indemnity.

Who has reviewed the trial?

All research in the NHS is looked at by an independent group of people, called a Research Ethics Committee (REC) to protect your safety, rights, well-being and dignity. This trial has been reviewed and given favourable ethical opinion by the London – Westminster Research Ethics Committee. It has also been approved by the local Research and Development department at your hospital.

Involvement of the General Practitioner/Family Doctor (GP)

As this trial requires you to take a drug which may cause side effects, it is important that your GP is informed that you are taking part in this trial. In addition, we may ask him/her to provide information on your progress. If we need to contact your GP for any follow-up information, we will need to use your full name in our correspondence.

2. Who should I contact for further information?

Further information and contact details

If you have any questions or concerns about your disease or this clinical trial, please discuss them with your doctor.

You may also find it helpful to contact the following free and independent organisations for confidential advice, support and information on healthcare matters:

England:

Patient Advice and Liaison Service (PALs)

https://www.nhs.uk/nhs-services/hospitals/what-is-pals-patient-advice-and-liaison-service/

Telephone: NHS 111

Northern Ireland:

Patient Client Council (PCC)

http://www.patientclientcouncil.hscni.net/

Telephone: 0800 917 0222

Scotland:

Patient Advice and Support Service

http://www.patientadvicescotland.org.uk/

Telephone: 0800 917 2127

Wales:

Community Health Councils (CHCs)

https://boardchc.nhs.wales/

Telephone: 02920 235 558

Other Sources of Information:

For more generic information about treating cancer, you may also find it helpful to contact '**About Cancer**', an information service about cancer and cancer research studies by Cancer Research UK:

Freephone: 0808 800 40 40

Website: www.cancerresearchuk.org/about-cancer/brain-tumours

For more information and support about brain tumours, you may find it helpful to contact **The Brain Tumour Charity**:

Freephone: 0808 800 0004 **Email:** support@thebraintumourcharity.org

Website: www.thebraintumourcharity.org/

Your telephone contact numbers:

Local Investigator: ....

Research Nurse: .... **Contact Number:** ....

Emergency (24 hours) Contact Number: ....

This completes Part 3 of the Information Sheet

Thank you for taking the time to read this Patient Information Sheet and considering taking part in this trial.

To be printed on hospital headed paper

ARISTOCRAT

A randomised controlled phase II trial of temozolomide with or without cannabinoids in patients with recurrent glioblastoma

Informed Consent Form

Site: ______________________ **Patient's Trial Number:** ☐☐☐

Principal Investigator: ______________________ **EudraCT Number:** 2021-005214-34

Please initial each box

1. I confirm that I have read and understand the Patient Information Sheet (version .... dated..........................) for the above trial. I have had the opportunity to consider the information, ask questions and have had these answered satisfactorily. ☐
2. I understand that my participation is voluntary and that I am free to withdraw at any time without giving any reason, without my medical care or legal rights being affected. ☐
3. I give my permission for my initials, date of birth, ethnicity, hospital number and NHS/CHI number to be given to the ARISTOCRAT Trial Office when I am randomised to the trial as well as a copy of this consent form. ☐
4. I understand that relevant sections of my medical notes and data collected during the trial may be looked at by individuals from the ARISTOCRAT Trial Office, regulatory authorities, Sponsors and/or NHS bodies, where it is relevant to my taking part in this research. I understand that this information will be held in a confidential manner. I give permission for these individuals to have access to my records. ☐

5. I understand that identifiable data from the trial may be shared with other authorised researchers, including the Tessa Jowell BRAIN MATRIX (University of Birmingham) and the BRIAN databank run by The Brain Tumour Charity. I give permission for my identifiable data to be shared for this purpose. ☐
6. I understand that information about me (including my trial number, date of birth, ethnicity and sex) may also be provided to other 3rd parties (e.g. pharmaceutical companies and other academic institutions) for research, safety monitoring or licensing purposes. ☐
7. I agree to my GP being informed of my participation in this trial. ☐
8. I understand that the ARISTOCRAT Trial Office may access information held by national cancer registries and within national databases to keep in touch with me and to follow up on my health status. ☐
9. I agree to the collection of tissue from any of my previous biopsies and future tumour surgeries and consent for these samples to be analysed as part of research associated with this trial, to be used in biobanking and for future ethically approved studies. ☐
10. I agree to take part in the above trial. ☐

**The following are <u>optional</u> and will not affect entry into the trial, please initial in the relevant box:**	**No**	**Yes**
I would like to receive a copy of the lay summary results at the end of the trial.	☐	☐
I agree to be approached about consenting to the collection of additional samples at routine sample time points (such as regular blood tests), subject to future funding being secured.	☐	☐

____________________	____________________	____________________
**Name of patient**	**Date**	**Signature**
____________________	____________________	____________________
**Name of person taking consent**<br>You must have signed the<br>Site Signature & Delegation Log	**Date**	**Signature**

Appendix 5: Guidelines of toxicity management and dose reductions for temozolomide

Haematological toxicity

- Dose for cycle 2 should only be increased to 200mg/m^2 if absolute neutrophil count (ANC) > 1.5 x 10^9/L and platelet count > 100 x 10^9/L.
- For all cycles, dose should be delayed until ANC > 1.5 x 10^9/L and platelet count > 100 x 10^9/L.
- Following delays for haematological toxicity dose should be reduced by 50mg/m^2 on subsequent cycles.
- Following dose reduction for toxicity no re-escalation should be undertaken in subsequent cycles.
- If toxicity occurs at dose of 100mg/m^2 or following two dose reductions, then temozolomide should be stopped.

Non-haematological toxicity

- Defer temozolomide dosing by 1 week for non-haematological Grade 3 toxicity.
- Consider dose reduction by 50mg/m^2.
- Discontinue temozolomide if Grade 3 toxicity at dose of 100mg/m^2.

Appendix 6: Definition of adverse events

Adverse Event (AE)

Any untoward medical occurrence in a patient or clinical trial subject administered a medicinal product and which does not necessarily have a causal relationship with this treatment.

Comment:

An AE can therefore be any unfavourable and unintended sign (including abnormal laboratory findings), symptom or disease temporally associated with the use of an investigational medicinal product, whether or not related to the investigational medicinal product.

Adverse Reaction (AR)

All untoward and unintended responses to an IMP related to any dose administered.

Comment:

An AE judged by either the reporting Investigator or Sponsor as having causal relationship to the IMP qualifies as an AR. The expression reasonable causal relationship means to convey in general that there is evidence or argument to suggest a causal relationship.

Serious Adverse Event (SAE)

Any untoward medical occurrence or effect that at any dose:

- Results in death unrelated to original cancer
- Is life threatening*

- Requires hospitalisation** or prolongation of existing inpatients' hospitalisation
- Results in persistent or significant disability or incapacity
- Is a congenital anomaly/birth defect
- Or is otherwise considered medically significant by the Investigator***

Comments:

The term severe is often used to describe the intensity (severity) of a specific event. This is not the same as serious, which is based on patients/event outcome or action criteria.

* Life threatening in the definition of an SAE refers to an event in which the patient was at risk of death at the time of the event; it does not refer to an event that hypothetically might have caused death if it were more severe.

**Hospitalisation is defined as an unplanned, formal inpatient admission, even if the hospitalisation is a precautionary measure for continued observation. Thus, hospitalisation for protocol treatment (e.g. line insertion), elective procedures (unless brought forward because of worsening symptoms) or for social reasons (e.g. respite care) are not regarded as an SAE.

*** Medical judgment should be exercised in deciding whether an AE is serious in other situations. Important AEs that are not immediately life threatening or do not result in death or hospitalisation but may jeopardise the subject or may require intervention to prevent one of the other outcomes listed in the definition above, should be considered serious.

Serious Adverse Reaction (SAR)

An Adverse Reaction which also meets the definition of a Serious Adverse Event.

Suspected Unexpected Serious Adverse Reaction (SUSAR)

A SAR that is unexpected i.e., the nature, or severity of the event is not consistent with the applicable product information.

A SUSAR should meet the definition of an AR, UAR and SAR.

Unexpected Adverse Reaction

An AR, the nature or severity of which is not consistent with the applicable product information (e.g., Investigator Brochure for an unapproved IMP or (compendium of) Summary of Product Characteristics (SPC) for a licensed product).

When the outcome of an AR is not consistent with the applicable product information the AR should be considered unexpected.
